# Supplementary material for: Accurate detection of identity-by-descent segments in human ancient DNA
Source: Nat Genet. 2023 Dec 20;56(1):143–51. doi: 10.1038/s41588-023-01582-w (PMC10786714; doi:10.1038/s41588-023-01582-w)
Supplement: Supplementary file 1 — Supplementary Notes 1–11. [file 41588_2023_1582_MOESM1_ESM.pdf]

nature genetics

Article

https://doi.org/10.1038/s41588-023-01582-w

Accurate detection of identity-by-descent segments in human ancient DNA

In the format provided by the authors and unedited

Supplementary Materials

October 9, 2023

Contents

Supplementary Note 1 **Emission Probabilities for IBD states** **2**

Supplementary Note 2 **Simulating IBD segment Data** **3**

Supplementary Note 2.1: Validation of Simulated IBD segment Data 5

Supplementary Note 3 **Imputation Pipeline** **9**

Supplementary Note 4 **Setting Allele Frequencies for ancIBD** **10**

Supplementary Note 5 **Downsampling Empirical aDNA Data** **11**

Supplementary Note 6 **Performance of ancIBD when using all common 1000 Genome SNPs** **16**

Supplementary Note 7 **Estimating False Positive Rates with Downsampled Empirical Data** **18**

Supplementary Note 8 **Simulating IBD-sharing of biological relatives using PED-SIM** **23**

Supplementary Note 9 **Identifying relatives using pair-wise mismatch rates (PMR)** **24**

Supplementary Note 10 **Comparison with other methods to detect IBD segments** **26**

Supplementary Note 11 **Other Supplementary Figures** **31**

$\mathbf{g}$	$P(\mathbf{g})$	$P(\mathbf{g}\|D)$	$P(\mathbf{g}\|s=1)$
$(\mathbf{0},0,\mathbf{0},0)$	$(1-p)^4$	$(1-x_{1A})(1-x_{1B})(1-x_{2A})(1-x_{2B})$	$(1-p)^3$
$(\mathbf{0},1,\mathbf{0},0)$	$p(1-p)^3$	$(1-x_{1A})x_{1B}(1-x_{2A})(1-x_{2B}))$	$p(1-p)^2$
$(\mathbf{1},0,\mathbf{1},0)$	$p^2(1-p)^2$	$x_{1A}(1-x_{1B})x_{2A}(1-x_{2B})$	$p(1-p)^2$
$(\mathbf{1},1,\mathbf{1},0)$	$p^3(1-p)$	$x_{1A}x_{1B}x_{2A}(1-x_{2B})$	$p^2(1-p)$
$(\mathbf{0},0,\mathbf{0},1)$	$p(1-p)^3$	$(1-x_{1A})(1-x_{1B})(1-x_{2A})x_{2B}$	$p(1-p)^2$
$(\mathbf{0},1,\mathbf{0},1)$	$p^2(1-p)^2$	$(1-x_{1A})x_{1B}(1-x_{2A})x_{2B}$	$p^2(1-p)$
$(\mathbf{1},0,\mathbf{1},1)$	$p^3(1-p)$	$x_{1A}(1-x_{1B})x_{2A}x_{2B}$	$p^2(1-p)$
$(\mathbf{1},1,\mathbf{1},1)$	p^4	$x_{1A}x_{1B}x_{2A}x_{2B}$	p^3

Table S1: List of ingredients for calculating emission probability for $s=1$

Supplementary Note 1 Emission Probabilities for IBD states

Here we compute the HMM emission probability $P(D|s)$ for the first IBD state ($s=1$). Due to symmetry, the emission probabilities for all other IBD states ($s=2,3,4$) can be calculated analogously by simple rearrangement.

First, we consider all possible combinations of phased genotypes that are compatible with $s=1$. The IBD state $s=1$ encodes haplotypes $1A,1B$ as being IBD, thus the alleles on haplotype $1A,1B$ have to match and be both reference or alternative. A total of $2\times2\times2=8$ genotype configurations are compatible with $s=1$. These eight configurations with their corresponding $P(\mathbf{g}),P(\mathbf{g}|D),P(\mathbf{g}|s=1)$ are listed in Tab. S1. The other eight configurations are not possible, and we have $P(\mathbf{g}|s=1)=0$ for those.

Therefore, summing over all possible genotype combinations $\mathbf{g}\in\{0,1\}\times\{0,1\}\times\{0,1\}\times\{0,1\}$, we obtain now:

$$
\begin{aligned}
P(D|s=1) \sim & \sum_{\mathbf{g}\in\mathcal{G}} \frac{P(\mathbf{g}|D)}{P(\mathbf{g})} P(\mathbf{g}|s=1) = \\
& \frac{(1-x_{1A})(1-x_{1B})(1-x_{2A})(1-x_{2B})}{1-p} + \frac{(1-x_{1A})x_{1B}(1-x_{2A})(1-x_{2B})}{1-p} \\
& + \frac{x_{1A}(1-x_{1B})x_{2A}(1-x_{2B})}{p} + \frac{x_{1A}x_{1B}x_{2A}(1-x_{2B})}{p} \\
& + \frac{(1-x_{1A})(1-x_{1B})(1-x_{2A})x_{2B}}{1-p} + \frac{(1-x_{1A})x_{1B}(1-x_{2A})x_{2B}}{1-p} \\
& + \frac{x_{1A}(1-x_{1B})x_{2A}x_{2B}}{p} + \frac{x_{1A}x_{1B}x_{2A}x_{2B}}{p} \\
& = \frac{(1-x_{1A})(1-x_{2A})(1-x_{2B})}{1-p} + \frac{x_{1A}x_{2A}(1-x_{2B})}{p} + \frac{(1-x_{1A})(1-x_{2A})x_{2B}}{1-p} + \frac{x_{1A}x_{2A}x_{2B}}{p} \\
& = \frac{x_{1A}x_{2A}}{p} + \frac{(1-x_{1A})(1-x_{2A})}{1-p}
\end{aligned}
$$

Supplementary Note 2 Simulating IBD segment Data

To test our method, we generated simulated genetic data with two key objectives in mind. First, we want to have accurate ground-truth IBD segments with exactly defined boundaries. Second, we want to mimic errors and imputation uncertainties of typical aDNA data, such as those caused by low coverage and postmortem damage.

Towards these two goals, we used the 1000 Genomes Phase 3 release [Consortium et al., 2015] and applied a two-step simulation procedure that is sketched in Supplementary Fig.1. The first step establishes synthetic individuals with perfectly known diploid phased genotypes and ground-truth IBD segments. The second step then adds the uncertainties of imputing low-coverage aDNA data by matching imputation inaccuracies that we established by imputing low-coverage versions of high-coverage aDNA data. Throughout, we simulate data for 1240k bi-allelic SNP sites, which are the input to ancIBD.

In the first step, we simulated diploid genotypes by creating a mosaic of haplotypes with the TSI group label (Tuscany, Italy) in the 1000 Genomes data following a previous approach [Browning and Browning, 2011, Ralph and Coop, 2013]. We copied TSI haplotypes in blocks of 0.25 cM length, where each block was chosen randomly from all TSI samples. The motivation for this haplotype mosaic approach is that any background IBD much longer than 0.25 cM that might exist between the 1000 Genomes TSI haplotypes is most likely broken up, while fine-scale background LD patterns are mostly maintained. We note that naturally recombining genomes similarly produce haplotype mosaics, only with not as regularly spaced switch points. We then grouped pairs of mosaic haplotypes into diploid genomes. To create ground-truth IBD blocks, we overwrote one of the haplotypes of a pair of diploid samples with the matching haplotype of the other sample. The start and end point of the overwrite was chosen randomly along the simulated chromosome, with the length of the overwrite matching specified IBD segment lengths. Using this approach, we simulated IBD segments 4,8,12,16,20 cM long, each with 500 replicates of pairs of diploid chromosomes 3.

In the second step, we added genotype probabilities and phasing errors to the mosaic genomes with added IBD segments, aiming to mimic errors introduced in the imputation process of empirical low-coverage aDNA data. Generally, imputation accuracy at SNPs depends on allele frequencies, and homozygotes are better imputed than heterozygotes [e.g. Hui et al., 2020, Sousa da Mota et al., 2023]. To estimate these complex dependencies, we downsampled and imputed 52 high-coverage ancient samples (50 of them >15x average coverage, and two >10x, all double-stranded library and half-UDG treated, Supp Table H) from AGDP (see data availability) to various target coverages. For each of those

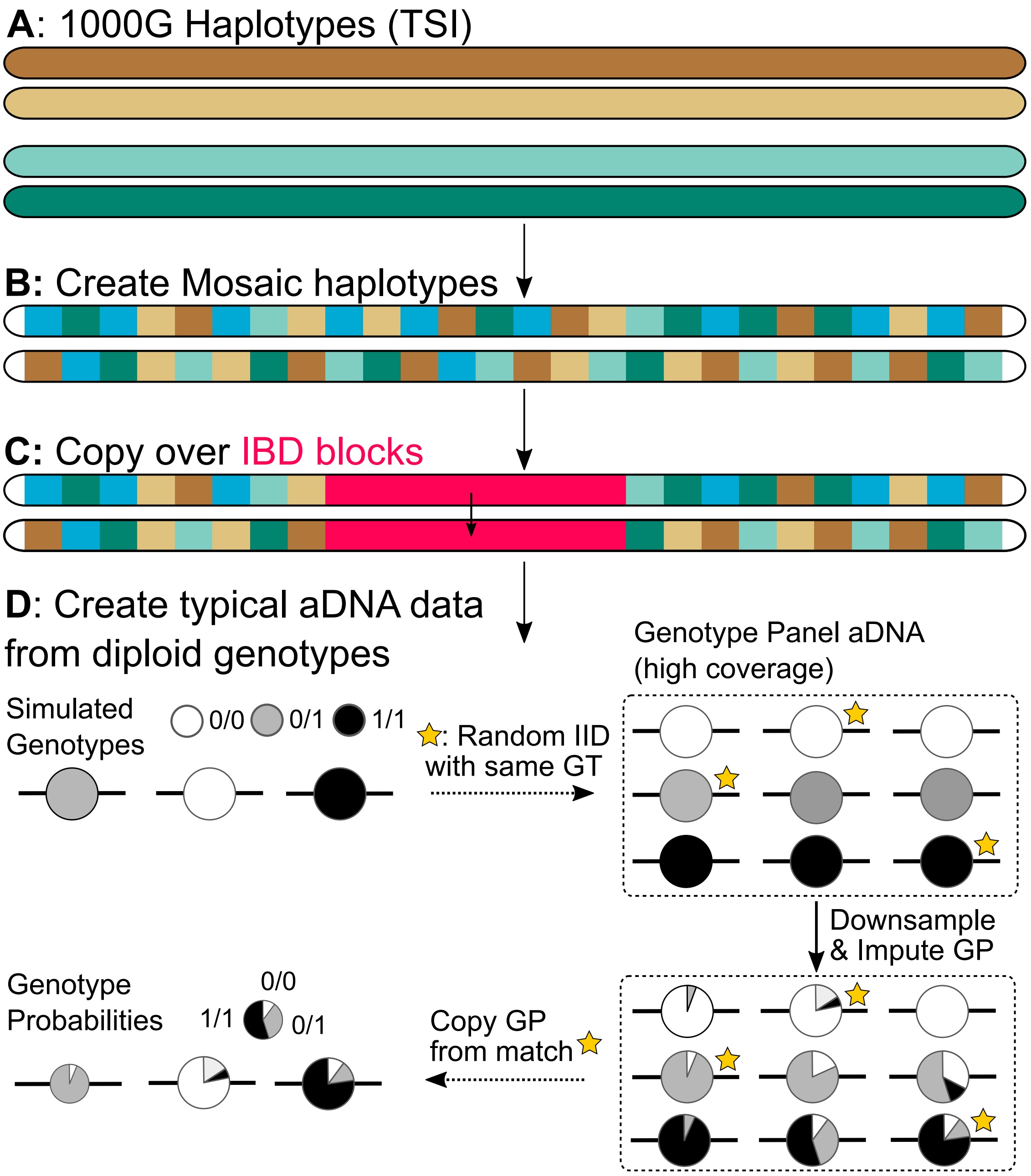

Supplementary Fig.1: Pipeline to simulate IBD segment data. We visualize our steps to simulate IBD segment data (see detailed description in Supplementary Note 2). Starting from TSI (Tuscany) high-quality reference haplotypes in the 1000 Genome panel (**A**), we created haplotype mosaics (**B**) as any long IBD segment is removed from those. We then copied over IBD segments of the target length (**C**). We grouped two mosaic haplotypes to obtain diploid individuals but to simplify visualization here we do not depict the second haplotype per individual. **D**: To create data typical for imputed low-coverage aDNA, we matched each genotype to a random matching genotype in a panel of aDNA diploid genotypes called from high-coverage aDNA (either 1240k or WGS aDNA data). We then downsampled the high-coverage aDNA panel to the target coverage, imputed genotype probabilities, and copied those back to each match.

52 individuals, both WGS and a 1240k captured aDNA data are available, and we used those separately to establish imputation inaccuracies for WGS and 1240k data, respectively. First, we established ground-truth genotypes for each individual by imputing the

original high-coverage WGS data after clipping 5 base pairs from both ends of aligned sequencing reads to reduce aDNA damage. For various target coverages and for each of the three possible genotypes (0/0, 0/1, 1/1) at each site, we then assembled a list of imputed genotypes and their associated genotype probabilities from the downsampled and imputed data. We then simulated imputation error by setting the genotype and genotype probability at each SNP of the mosaic genomes to those of a sample chosen randomly from the aforementioned list of imputed ancient genomes with the same true genotype and target coverage. In case a genotype is not found in any of the 52 high-coverage samples, we kept the true simulated genotype and set its associated genotype probability to 99%. We note that of all 77,652 biallelic 1240k markers on chromosome 3, 52,629 have all three possible genotypes found in at least one of the 52 genomes, and 15,700 of them have two of the three possible genotypes.

Finally, we introduced phasing errors by flipping the phase at intervals drawn from an exponential distribution. To specify this distribution, we matched the average phased block length estimated from downsampling a high-quality trio (I3388, I3950, I3949, whose high-coverage WGS data were published in Wohns et al. [2022] and 1240k data in Narasimhan et al. [2019]). Both WGS and 1240k BAM files of this trio set were downsampled to 2x, 1x, 0.75x, 0.5x, 0.25x, 0.1x and then imputed and phased with GLIMPSE as described in Supplementary Note 5. We identified phase switches between 1240k SNPs using VCFtools –diff-switch-error [Danecek et al., 2011]. The average phase block lengths for WGS and 1240k are summarized in Tab. S2.

Supplementary Note 2.1: Validation of Simulated IBD segment Data

Our approach effectively combines SNP genotype probabilities from a mixture of downsampled individuals. To validate whether our simulation procedure mimics empirical downsampled and imputed aDNA data we ran two sets of experiments.

First, we compared the distribution of max(GP) (defined as the maximum among the three posterior genotype probabilities of 0/0,0/1,1/1) between simulated individuals and that of downsampled and imputed empirical individuals. The overall fraction of max(GP)>0.99 among all imputed variants strongly correlates with coverage (Supplementary Fig.5); indicating that the distribution of max(GP) is a good indicator of imputation quality. Reassuringly, when comparing the cumulative distribution for simulated and empirical downsampled aDNA data, the CDFs match well both for 1240k and WGS data types (Supplementary Fig.2).

Second, we examined the number of SNPs being incorrectly imputed (i.e. the imputed

Coverage	WGS [cM]	1240k [cM]
**2x**	0.182	0.182
**1x**	0.257	0.125
**0.75x**	0.271	0.106
**0.5x**	0.271	0.0814
**0.25x**	0.220	0.0532
**0.1x**	0.127	0.0344

Table S2: Mean Phased Block Length for WGS and 1240k Data at Various Average Coverages. All lengths are map lengths measured in centimorgan. Phase switch errors are inferred as described in Supplementary Note 2 by downsampling a high-coverage ancient parent-offspring trio, using the high-coverage data as ground truth.

GT is different from the ground truth genotype). We computed the confusion matrix that describes the frequency of 0/0,0/1,1/1 genotypes to be imputed as 0/0,0/1,1/1 on 1240k SNPs on Chromosome 3 for both 1240k and WGS data types. Reassuringly, we found that the matrix of simulated data is nearly identical to that of empirical downsampled and imputed data (Supplementary Fig.3). This finding demonstrates that we introduce genotype errors at rates matching those observed for typical empirical ancient DNA.

Lastly, we stress that the phasing switch error that we introduced into the simulated data was the one we directly estimated from downsampled high-coverage trios (see Tab. S2). Therefore, by design, the average length of correctly phased segments in our simulated data matches those observed in low-coverage aDNA established when compared to the gold standard of trio phasing the high-coverage versions.

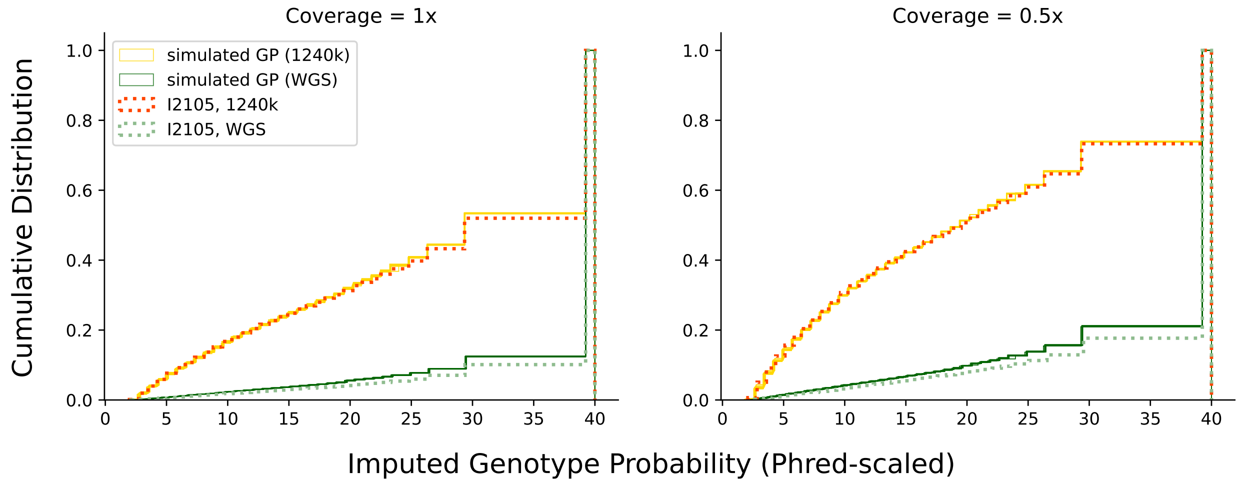

Supplementary Fig.2: Cumulative Distribution Function (CDF) of Maximum Genotype Posteriors at Each Loci. We compared the CDF of maxGP between simulated data and empirical downsampled data at 1x and 0.5x coverage, for both 1240k and WGS data types. The genotype probability is plotted on a phred-scale and capped at 40 (corresponding to 0.9999).

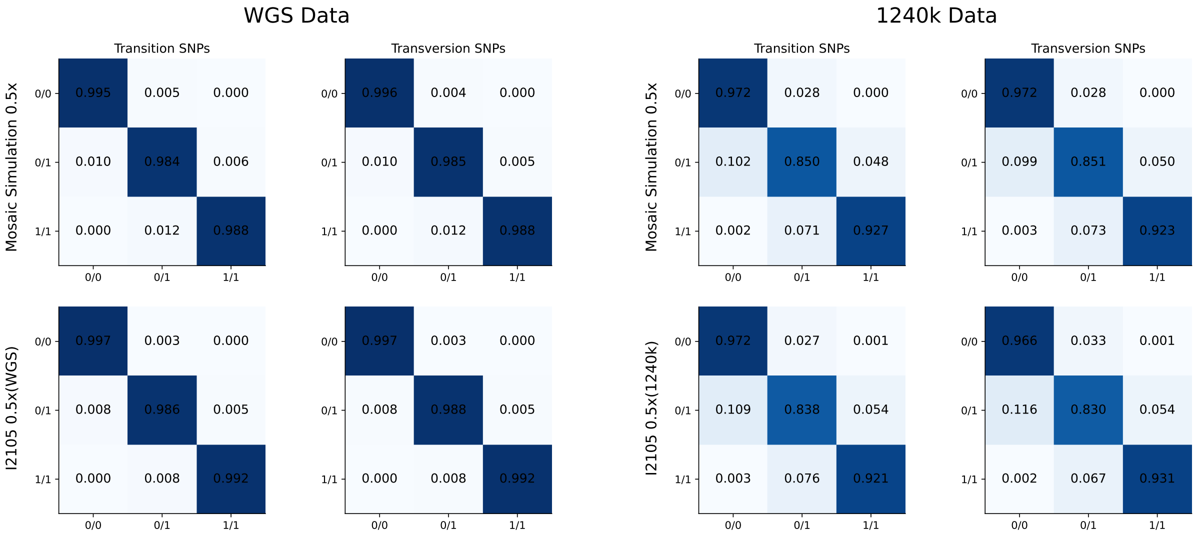

Supplementary Fig.3: Confusion Matrix of Imputed Genotypes for WGS and 1240k Data. Confusion matrix showing the probability of ground-truth genotype 0/0,0/1,1/1 being imputed as one of 0/0,0/1,1/1 at coverage 0.5x. We stratified results by SNP type (transition vs. transversion SNPs). The first row shows the confusion matrix in simulated data while the second row shows that in empirical downsampled aDNA data. The left panel depicts WGS data, and the right panel 1240k data.

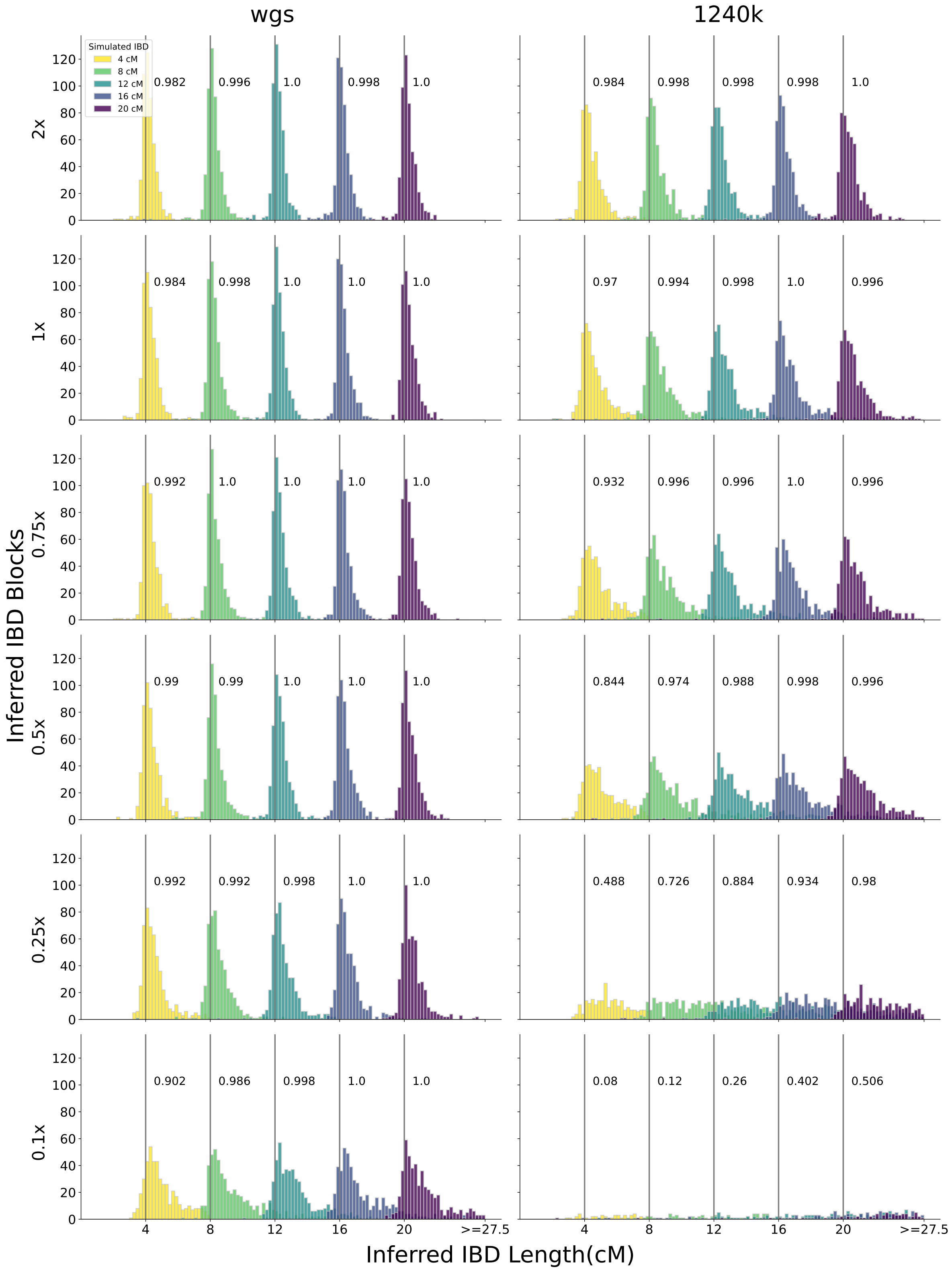

Supplementary Fig.4: IBD Calling Accuracy. Accuracy of IBD calling in simulated synthetic diploid samples with IBD segments of length 4,8,12,16,20 cM. We simulated shotgun-like and 1240k-like data as described in Supplementary Note 2). We visualize false positive, power, and general length bias for coverages from 2x down to 0.1x (rows). We indicate power to call segments of each simulated length next to the respective gray vertical bars.

Supplementary Note 3 Imputation Pipeline

In our downsampling experiments, we imputed aDNA with GLIMPSE following standard settings, as previously described in Waldman et al. [2022]: We first generated genotype probabilities using bcftools mpileup (v1.10.2) [Li, 2011] (with -q 30 -Q 30 filtering to use only high-quality aligned reads and bases). To generate the genotype likelihood VCF file as input for GLIMPSE, we used the following command:

```
bcftools mpileup -f ${REFGEN} --ignore-RG -I -E \
-a 'FORMAT/DP' -T ${VCF} -r $ch -q 30 -Q 30 ${BAM} -Ou | \
bcftools call -Aim -C alleles -T ${TSV} -Oz -o ${OUT}
```

Here, REFGEN refers to the human reference genome fasta file (here: build GRCh37/hg19), and the VCF and TSV files are generated from the 1000Genome Phase 3 reference panel, following the steps outlined in GLIMPSE tutorial (step 3.1 in https://odelaneau.github.io/GLIMPSE/glimpse1/tutorial_hg19.html). We note that, even for 1240k data, this genotype likelihood calling should also be performed on all 1000Genome bi-allelic SNPs as nearby SNPs also markedly improve the imputation accuracy of 1240k SNPs (Supplementary Fig.5). All code used for imputation is available at https://github.com/hyl317/IBDBenchmark.git. A total of 78,397,683 SNPs are used during imputation (only bi-allelic SNP sites from 1000 Genome are imputed). The 1240k SNP set consists of 1,100,313 SNPs after quality control and is widely used in aDNA studies. We note that it is a strict subset of the 1000G bi-allelic SNPs.

For the empirical applications of ancIBD described in this article, we started from the processed .bam files underlying the public Allen Ancient DNA Resource (AADR) [Mallick et al., 2023]. We generally recommend that the input .bam files are processed using standard aDNA processing, such as removing PCR duplicates, trimming of terminal base pairs to remove aDNA damage, and checking for contamination - see commonly used aDNA pipeline practices in Eager https://nf-co.re/eager, Yates et al. [2021]. We then imputed all autosomal bi-allelic SNPs 1000 Genomes Phase 3 release using GLIMPSE (v1.1.1), using its default parameters and following the recommended steps as described in the official GLIMPSE tutorial https://odelaneau.github.io/GLIMPSE/glimpse1/tutorial_hg19.html.

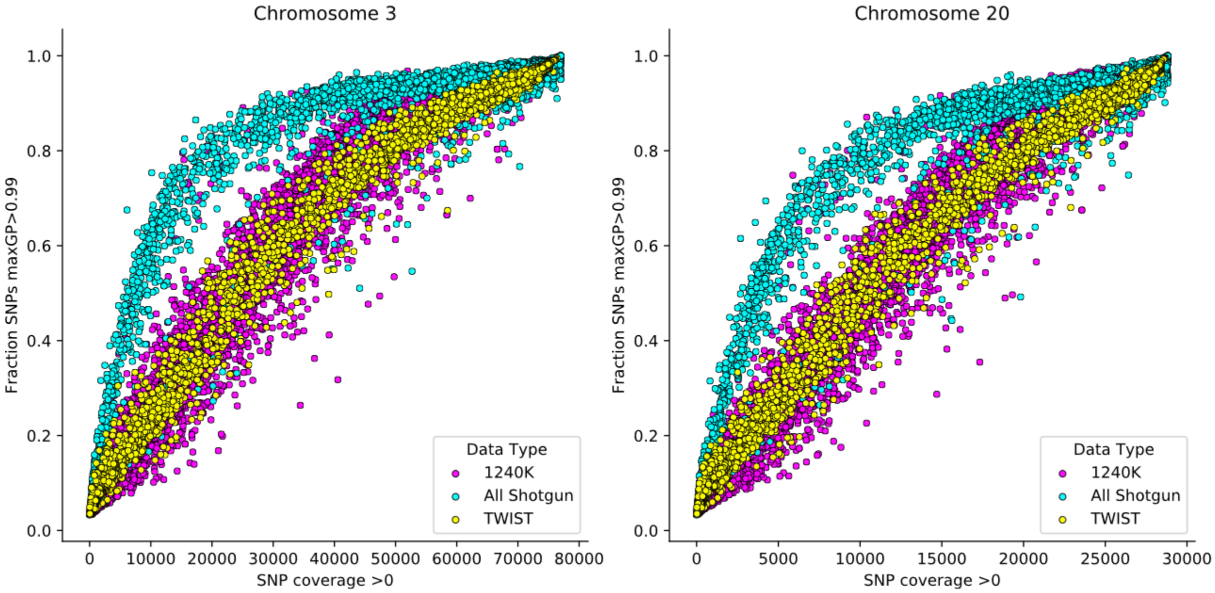

Supplementary Fig.5: Imputation Quality reported by GLIMPSE plotted against Coverage on 1240K SNPs. The two scatter plots visualize the imputation quality versus coverage for various ancient individuals. Each dot represents one individual. The x-axis marks the fraction of 1240K SNPs that are covered at least once by a sequencing read. The y-axis marks the fraction of 1240K SNPs where the highest imputed diploid genotype probability (GP field) is greater than 0.99, as provided by GLIMPSE. Note that imputation, as throughout, uses sequence data from all bi-allelic 1000G SNPs. We color-code individuals by their data type: 1240K and Twist capture, as well as shotgun sequencing data. We did this analysis for a typical long chromosome (chromosome 3, left plot) and a typical short chromosome (chromosome 20, right plot). Note that WGS data outperforms capture data of the same coverage - as shotgun data has also a large number of reads off-target that are evidently useful for imputation on 1240K SNPs.

Supplementary Note 4 Setting Allele Frequencies for ancIBD

To identify IBD with ancIBD, one has to specify allele frequencies in its emission model. Throughout this work, we used the allele frequencies of the 1000 Reference panel, which is output by GLIMPSE imputed data in the field INFO/RAF of the output VCF and is automatically transferred to the hdf5 file that is used as input to ancIBD. However, we note that ancIBD allows the user to specify other allele frequencies, and we provide the user a function to integrate allele frequencies into the hdf5 file (lift_af_df), and also an option to calculate the allele frequency of the imputed sample that is saved into the field AF_SAMPLE (which then can be used when running ancIBD). Moreover, users can also specify the allele frequency via a separate file when running ancIBD on the command line.

The dependency of ancIBD on allele frequency settings is expected to be relatively minor, as allele frequencies of common SNPs are relatively similar, even on continental levels (see e.g. [Biddanda et al., 2020], also Supplementary Fig.6). To test this intuition, we ran ancIBD on the whole empirical dataset using the allele frequencies of the imputed Eurasian data (consisting only of ancient DNA samples from Eurasia) - calculated when using SNPs from imputed individuals only where the maximum genotype probability

was bigger than 0.99. Reassuringly, we find that the IBD calls on the empirical dataset correlate closely (see Supplementary Fig.6). Generally, we recommend using the allele frequencies of the 1000G reference panel, as these are calculated from a sufficient set of samples and have been tested well throughout this work.

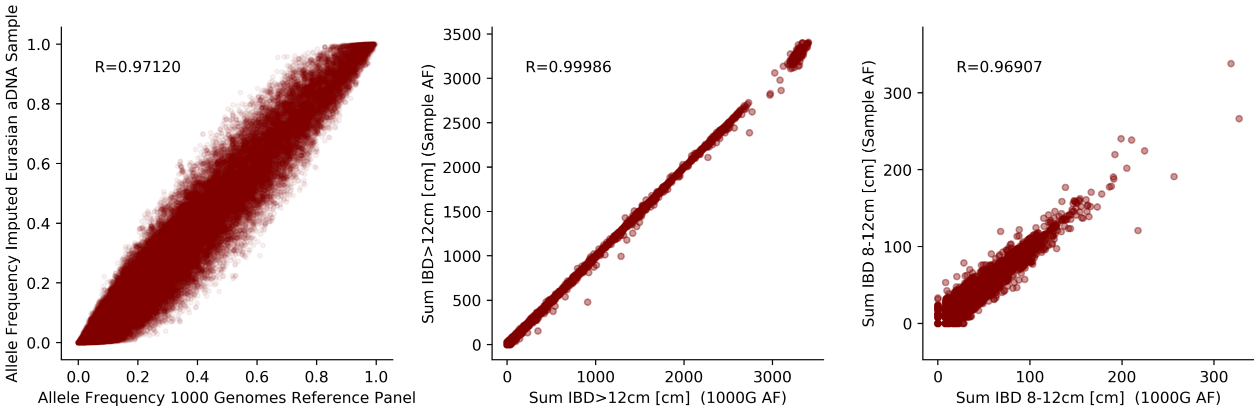

Supplementary Fig.6: ancIBD calls on empirical dataset when using different allele frequencies. We ran ancIBD on the whole empirical dataset consisting of 11,404 ancient genomes, using either allele frequencies from the 1000G reference panel or calculated on the full imputed data (see scatter plot left that compares those two allele frequencies for each SNP). Using the called IBD segments of the two large ancIBD runs, we visualize a comparison of the sum per pair of all IBD longer than 12 cM (middle), as well as the sum of all IBD 8-12 cM long (right), each dot corresponds to one pair of individuals and all sums refer to values in cM. We also depict the Pearson product-moment correlation (Pearson's R, see text labels upper left).

Supplementary Note 5 Downsampling Empirical aDNA Data

To assess the performance of ancIBD on realistic aDNA data, we downsampled high-coverage (∼20x) empirical human ancient DNA data. To obtain ground truth IBD segments to compare to, we used four WGS samples associated with the Afanasievo culture: I2105 (23.0X, 3300-2500 BCE, Ukraine, [Mathieson et al., 2018]); I3950 (25.8x, 2879-2632 calBCE, Russia, [Narasimhan et al., 2019]); I5273 (22.4x, 3011-2885 calBCE, [Narasimhan et al., 2019]) and I5279 (28.4x, 3011-2897 calBCE, Russia, [Narasimhan et al., 2019]).

To establish ground-truth diploid genotypes for those four samples, we computed genotype likelihoods from the high-coverage BAM files using bcftools and then applied GLIMPSE to impute diploid genotypes. We then filtered to transversion sites and called IBD segments with IBIS [Seidman et al., 2020]. This algorithm takes as input unphased diploid genotypes and utilizes the fact that in the absence of genotyping error, two samples cannot be homozygous for two different alleles (”opposing homozygotes”) within an IBD region as the two samples have one of their haplotypes identical. This signal establishes a necessary condition for IBD, and the absence of opposing homozygotes over a long genomic region constitutes distinct evidence for IBD. The reason we chose IBIS to

establish ground truth IBD segments is that very few high-coverage trio samples are available for aDNA and computational phasing with 1000 Genomes reference panel produces relatively high switch error rates (Tab. S2, [Sousa da Mota et al., 2023]). After restricting to transversion biallelic sites and applying a posterior $GP > 99\%$ and $MAF > 1\%$ (minor allele frequency) filters, we obtained 3,756,564 sites for IBD screening. IBIS identified a total of 157 IBD segments longer than 4 cM among the six pairs of the four samples. We visually inspected those detected IBD segments to confirm that they are depleted of opposing homozygotes and removed 23 of them that contained regions with very low SNP density, typically segments located over centromeres or on chromosome ends. We used the resulting 134 segments as ground-truth IBD blocks for benchmarks discussed in the following.

For the four Afanasievo samples, both WGS and 1240k capture data are available. We downsampled the WGS BAM files to 2x, 1x, 0.75x, 0.5x, 0.25x and 0.1x coverage and the 1240k BAM files to 2x, 1x, 0.75x and 0.5x, each with 50 replicates. We applied the same bcftools+GLIMPSE imputation pipeline as described in Supplementary Note 3 and then ran ancIBD using its default parameters. We computed the precision and recall of ancIBD at various length bins and coverages when compared to the ground-truth IBD set described above. Similarly, we also screened the downsampled data with IBIS, using the same 1240k SNP set. Within a given map length bins of [5cM, 6cM), [6cM, 8cM), [8cM, 12cM), and >12cM, we calculated precision as the fraction of all inferred IBD segments that have at least 50% of their length covered by any true segment of any size and recall as the fraction of the total length of all true IBD segments that are at least 50% covered by inferred IBD segments of any size. Our results are summarized in Supplementary Fig.7.

Most notably, we found that for the same coverage, WGS data substantially outperforms 1240k data. Particularly, we found that 0.25x WGS data yields similar IBD calling accuracy as 1-2x 1240k data, both for ancIBD and IBIS.

For long IBD segments (>12cM) that are of particular interest for detecting relatives, ancIBD achieves both high precision and recall ($>90\%$) for all coverages tested here. Errors for segments in these length ranges remain negligible for most downstream analyses. We find that IBIS has substantially reduced power to identify IBD at lower coverages (<0.25x for WGS and <1x for 1240k), despite maintaining a consistently high precision over all coverages. For intermediate range segments (8-12cM), IBIS maintains relatively high precision (>90%) at all coverages tested while having reduced power at low coverages. ancIBD maintains high recall (∼80%) at all coverages while having less than 80% precision at 0.5x for 1240k data. Overall, our results demonstrate that ancIBD yield accurate IBD calling (∼90% or higher precision) at >0.25x WGS and >1x 1240k data.

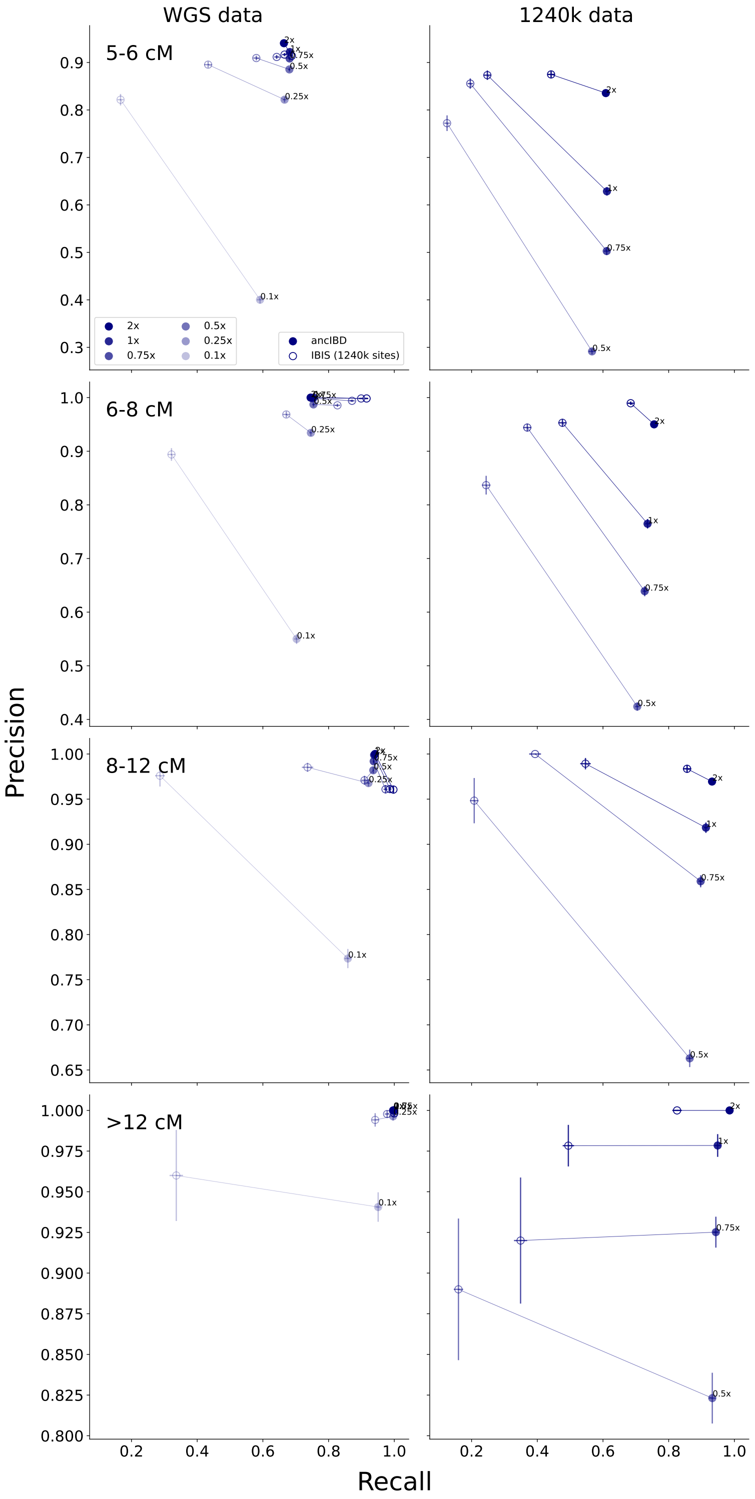

Supplementary Fig.7: Precision and recall of ancIBD and IBIS at various length bins and coverages. We applied both methods with their default settings to genotype data imputed after downsampling to various coverages. For each coverage, we report the average precision and recall of each length bin across 50 independent replicates. The error bar represents ±SE of the estimated precision and recall. Each row represents a length bin and each column represents one input data type (either WGS data or 1240k data). Note that the y-axis ranges are different for different rows.

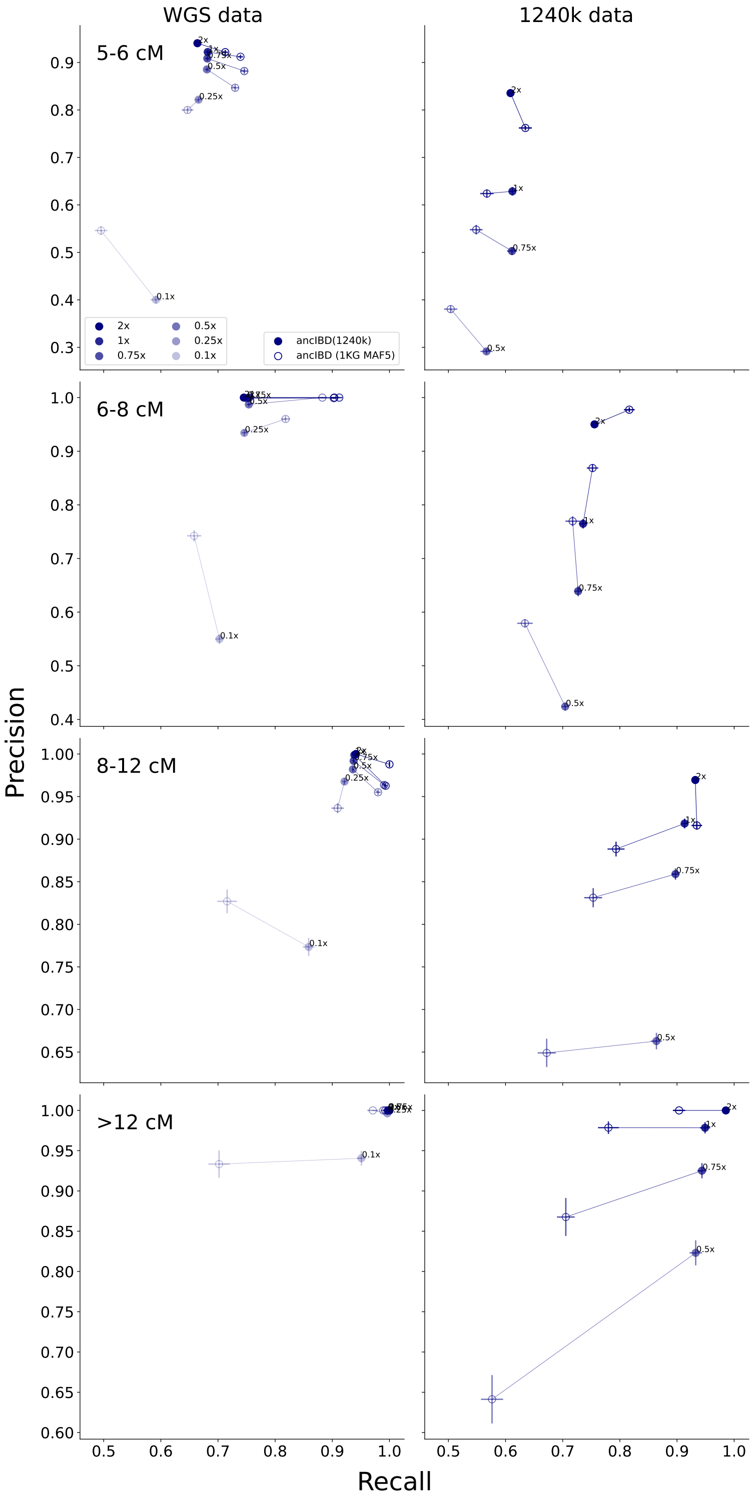

Supplementary Fig.8: Comparing precision and recall of ancIBD when using 1240k and 1000G MAF5 SNP sets. We applied ancIBD to either 1240k SNP and 1000G SNP filtered by minor allele frequency 5%. For 1KG MAF5 SNPs, we increased the parameter snp_cm to 800 to account for the higher density of SNPs in this larger SNP set. For each coverage, we report the average precision and recall of each length bin across 50 independent replicates. The error bar represents ±SE of the estimated precision and recall.

Our results indicate that for studies using shorter IBD segments (6-8 cM), which are often a main signal for demographic inference, greater care should be taken as false positive rates and false negatives are not trivial anymore. The default SNP density filtered (as described in the method section of the main article) reduces the ancIBD's recall for these shorter segments (~65-70%). To improve the performance of ancIBD, we designed genomic masks that filter IBD in regions prone to false positive IBD segments due to low SNP density (Supplementary Fig.9). To identify regions with excessive IBD sharing, we computed the average IBD sharing rate (>6cM) among 10,156 Eurasian ancient individuals (same set as in Fig. 3) in genomic windows of size 0.5 cM. We then designated regions to be masked as those whose sharing rate exceeds three standard deviations from the genome-wide average IBD sharing. The start and end point of each masked region was determined by the first windows (on the left and right) whose sharing rate equals or falls below the genome-wide average. With the mask applied, the precision of ancIBD without SNP density filtering remains as high as the one without mask and with SNP density filtering; however, we observe a substantial boost in power within the unmasked region to greater than 90% (Supplementary Fig.10).

We note that the precision of ancIBD reported in those downsampling experiments should be interpreted as being conservative because we likely underestimate precision in our downsampling experiments for the following two reasons. First, our benchmarks indicate that IBIS prioritizes precision over recall, especially for shorter segments, as reported previously (Seidman et al. [2020], Fig. 3 and Fig. S4 therein). Thus, IBIS might miss some true IBD segments in the high-coverage data that are called by ancIBD in the downsampled data. Second, we visually screened all the detected IBD segments and as ground truth only retained those that are depleted of opposing homozygotes without major gaps, which might effectively remove some true IBD segments.

To assess whether some IBD inferred by ancIBD are missing in the ground truth data set, we computed the rate of ”opposing homozygotes” for each detected segment using the genotypes called from the high-coverage BAM files. We define the rate of opposing homozygotes as the percentage of sites where two samples carry homozygotes for different alleles out of all sites where both samples carry homozygotes. We included only transversion sites with minor allele frequency $>10\%$ in the 1000G reference panel in this calculation so that the probability of being homozygote for both reference and alternative alleles is non-negligible. We then plotted this rate of opposing homozygotes against a segment's Positive Predictive Value (PPV), defined as the fraction of a called segment covered by any segments in the ground-truth set. We found many segments with low PPV that have rates of opposing homozygotes similarly low as segments with very high

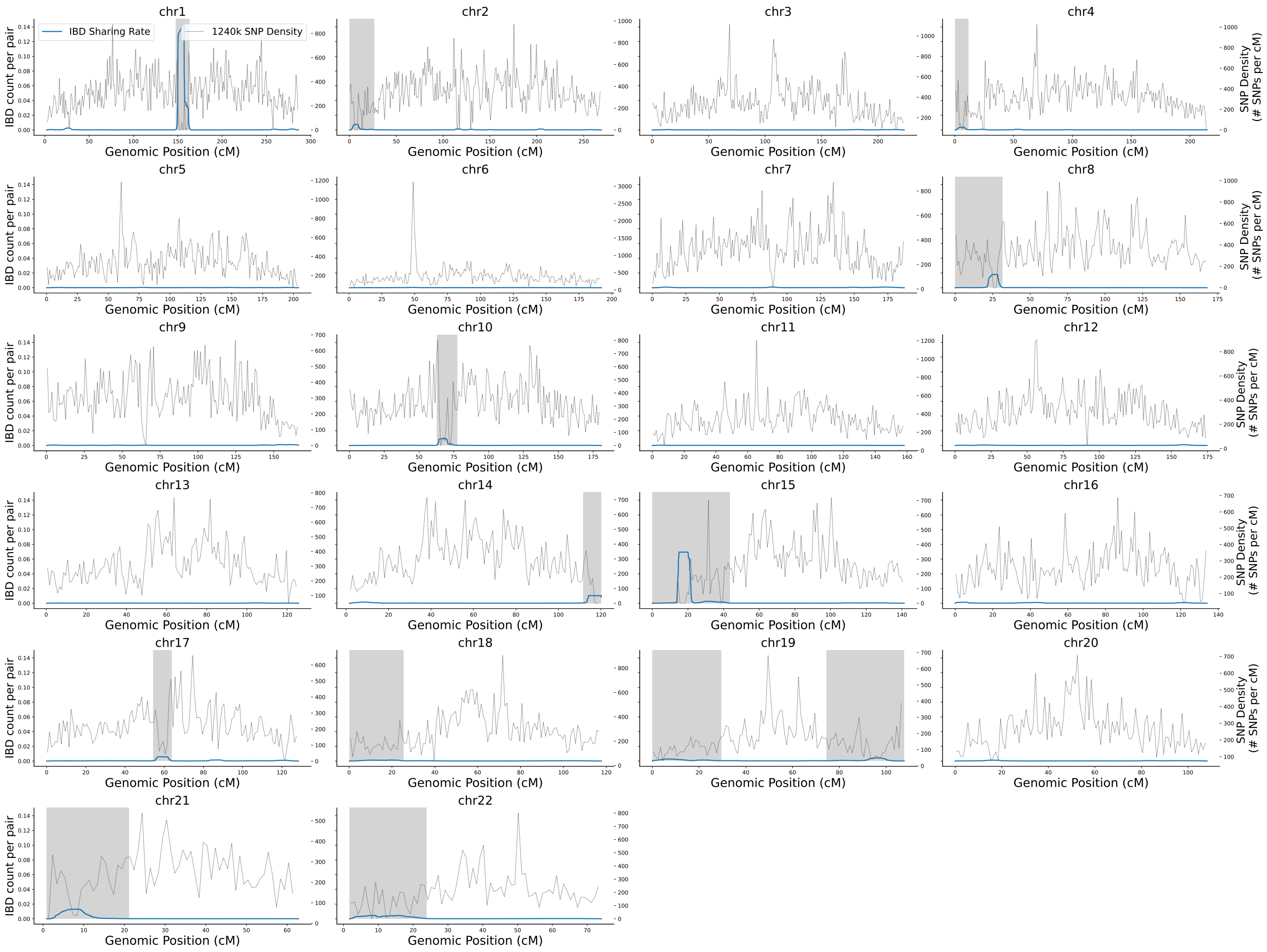

Supplementary Fig.9: IBD Sharing Rates along the genome. Average genome-wide IBD sharing rate on the 22 autosomes plotted for all the 1240k target sites. We indicate regions with excessive sharing of IBD that are excluded when using our mask (gray areas). The average sharing rate was computed from the IBD inferred between 10,156 ancient individuals described in the main manuscript.

PPV (Supplementary Fig.11, Supplementary Fig.12, Supplementary Fig.13, Supplementary Fig.14). This observation indicates that in the ground-truth set least parts of true IBD segments are missed, which would decrease the precision of ancIBD. That said, it is hard to determine whether these segments of exceptionally low opposing homozygote rates are fully true IBD segments. Thus we chose to be conservative in our tests.

Supplementary Note 6 Performance of ancIBD when using all common 1000 Genome SNPs

We explored whether using all common variants in the 1000 Genome variant sets can improve the performance of ancIBD. We filtered to 1000 Genome SNPs with minor allele

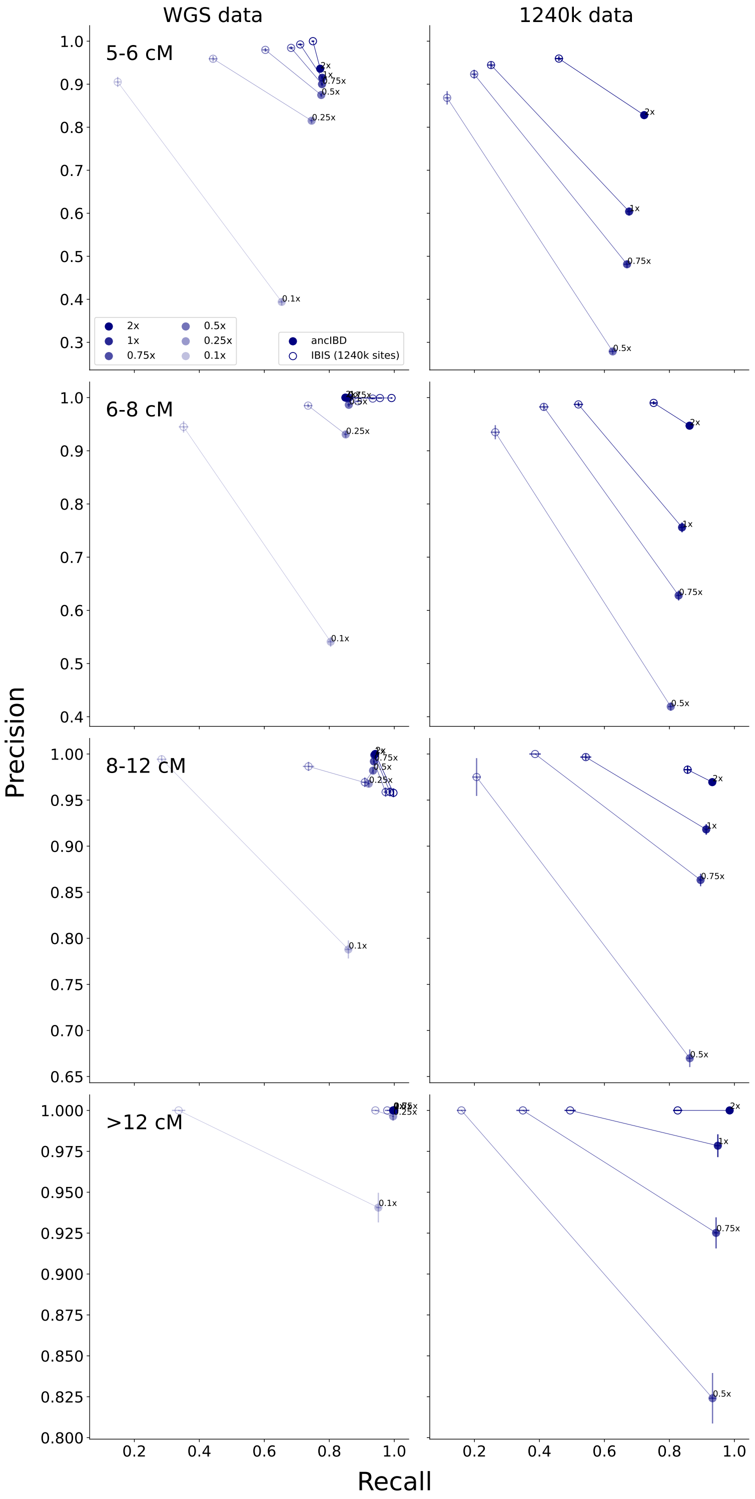

Supplementary Fig.10: Precision and recall of ancIBD and IBIS for various IBD length bins and depths of coverage. We applied ancIBD using the genomic masks (shown in Supplementary Fig.9) and without SNP density filtering. The error bar represents ±SE of the estimated precision and recall. All other settings are as in Supplementary Fig.7. For each coverage, we report the average precision and recall of each length bin across 50 independent replicates. The error bar represents ±SE of the estimated precision and recall.

frequency (MAF) greater than 5 percent and adjusted the parameter "snp_cm" (minimum SNP density per cM within IBD segments) from the default 220 to 800 because there are about six times more 1000G SNPs with MAF>5% than 1240k SNPs. We tested this expanded SNP set both on WGS and on 1240K aDNA data. For WGS aDNA data using 1000G SNPs with MAF>5% resulted in slightly improved performance for shorter IBD segments (6-8cM) while for long segments (>12cM) the precision and recall remain almost identical (Supplementary Fig.8). For 1240k aDNA data using 1000G SNPs with MAF>5% gave mixed results (Supplementary Fig.8). In particular, for longer segments (>8 cm), we observe a marked reduction in recall compared to when using 1240K SNPs.

As the benefits of utilizing all imputed common variants in 1000G SNPs are limited to WGS data and generally small, we recommend that ancIBD is run on data filtered to the 1240k SNP set after imputation. This has the practical benefit of increasing the co-analyzability of 1240k and WGS data, by having a single standard pipeline that can be applied to both kinds of data and also mixes thereof. However, we note that ancIBD can be run on any SNP set that is provided as input data, users can in principle choose and experiment with other SNP sets.

Supplementary Note 7 Estimating False Positive Rates with Downsampled Empirical Data

False positive IBD segments are particularly problematic for many downstream analyses such as demographic inference; therefore, it is important to establish for which coverage and IBD length cutoffs the false positive rate is tolerable for a particular application. To estimate false positive rates from empirical data, we selected 13 ancient individuals (I4893, I4596, I1583, I2978, I5838, I1507, I2861, I2520, I3758, I5077, I0708, I5233, I3123) from AGDP (see Data Availability and Supp. Tab. 1F) that have both high-coverage WGS and 1240k aDNA data available. All samples are chosen to be from Western Eurasia so that their imputation quality is expected to be relatively homogeneous and the estimated false positive rates are not driven by a subset of them being poorly imputed. We determined the ground-truth diploid genotypes on chromosome 3 as described in Supplementary Note 5 and then used IBIS to confirm that these samples share no IBD with each other. We further verified the absence of IBD sharing by plotting opposing homozygous along chromosome 3 for visual inspection. Therefore, all inferred IBD segments from the downsampled data are false positives.

For WGS data, we downsampled to 0.1x, 0.25x, 0.5x, and 0.75x. For 1240k data, we

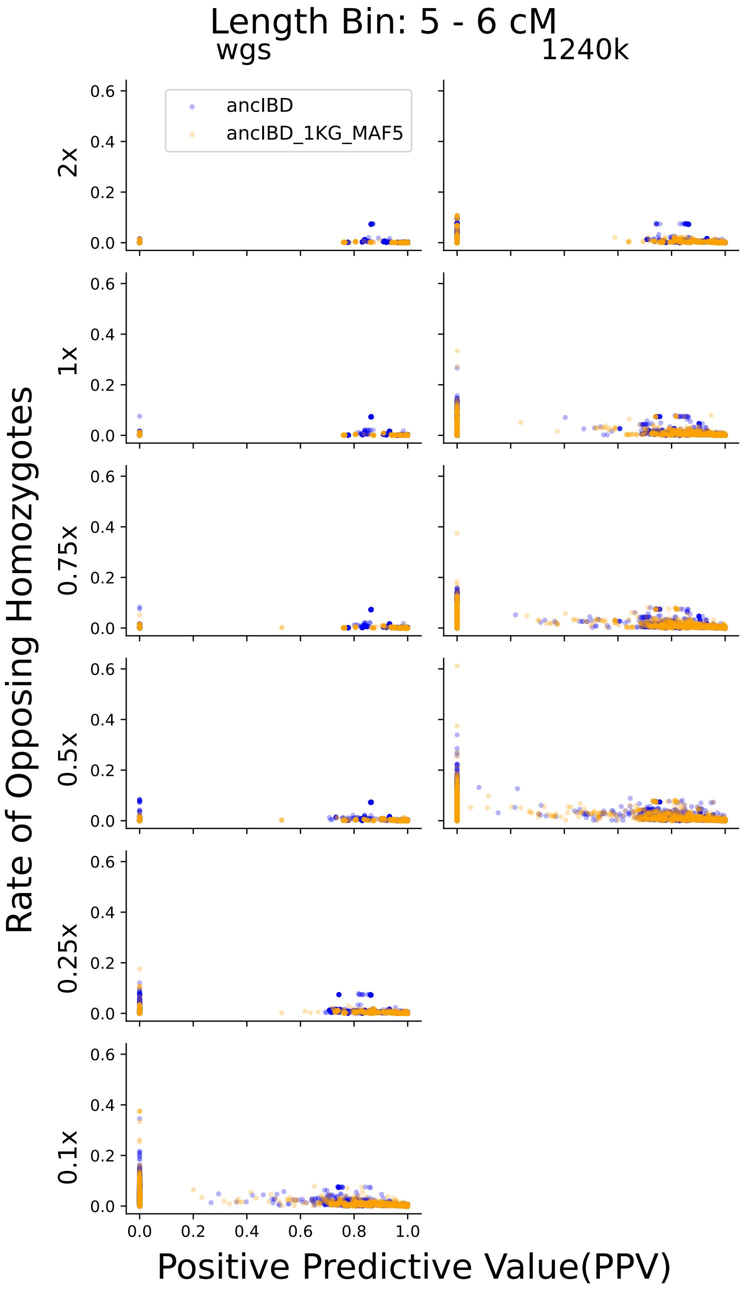

Supplementary Fig.11: Rate of Opposing Homozygotes for called segments in length bin 5-6cM.

downsampled to 1x, 2x, and 3x (for 3x downsampling, we excluded samples I5233 and I5077 due to insufficient coverage of the original 1240k BAM file). For each target coverage, we created 50 independent replicates and the estimated average false positive rates are visualized in Fig. 2b.

As in Supplementary Note 5, we found that WGS data outperforms 1240k aDNA data of the same coverage. We also note that, depending on different applications, the coverage cutoff for ancIBD is different. For example, for detecting biological relatives using

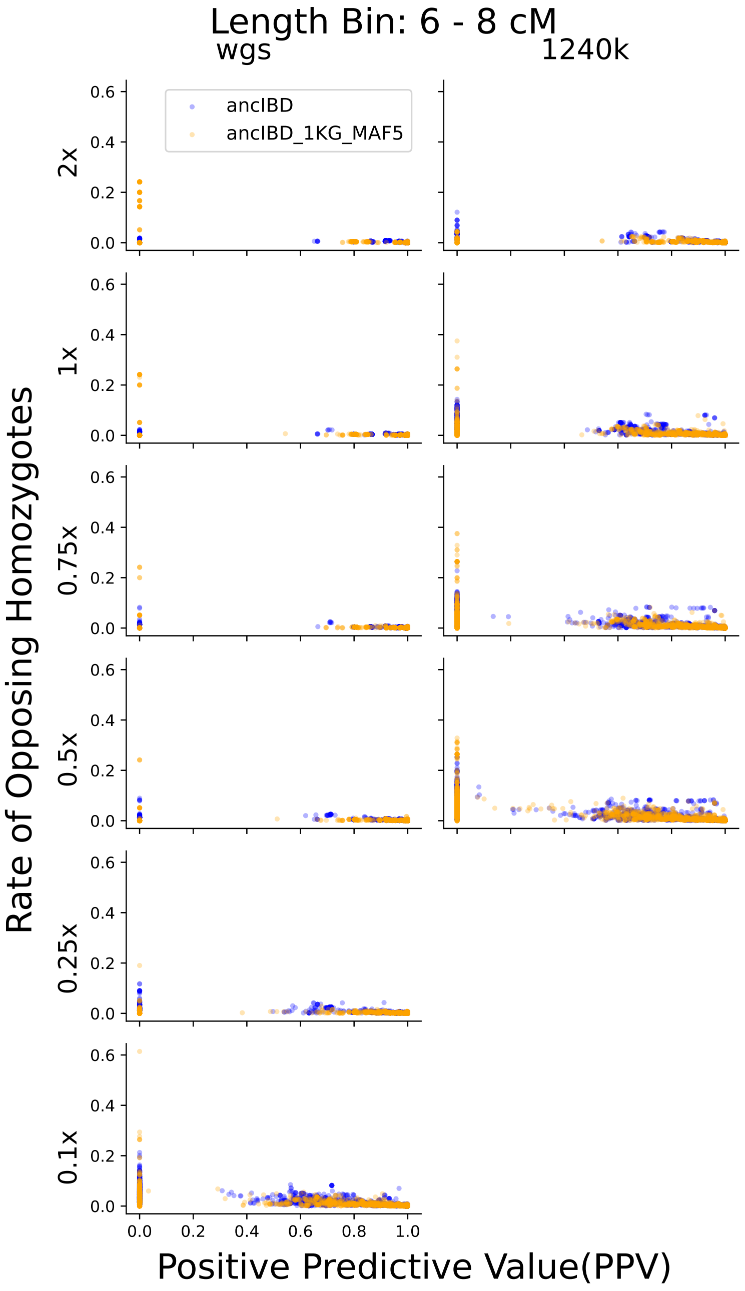

Supplementary Fig.12: Rate of Opposing Homozygotes for called segments in length bin 6-8cM.

IBD segments longer than 12cM, a more lenient coverage requirement can be employed (0.25x for WGS data and 1x for 1240k data). For demographic inference, one must take into account the signal-to-noise ratio; therefore, the IBD length and coverage cutoff are dependent on the effective population size of the study population and should be decided on a case-by-case basis. In Fig. 2b we plotted expected IBD sharing for constant populations with different effective population sizes to aid such comparison. To calculate this expected sharing, we use established formulas for expected IBD in panmictic populations

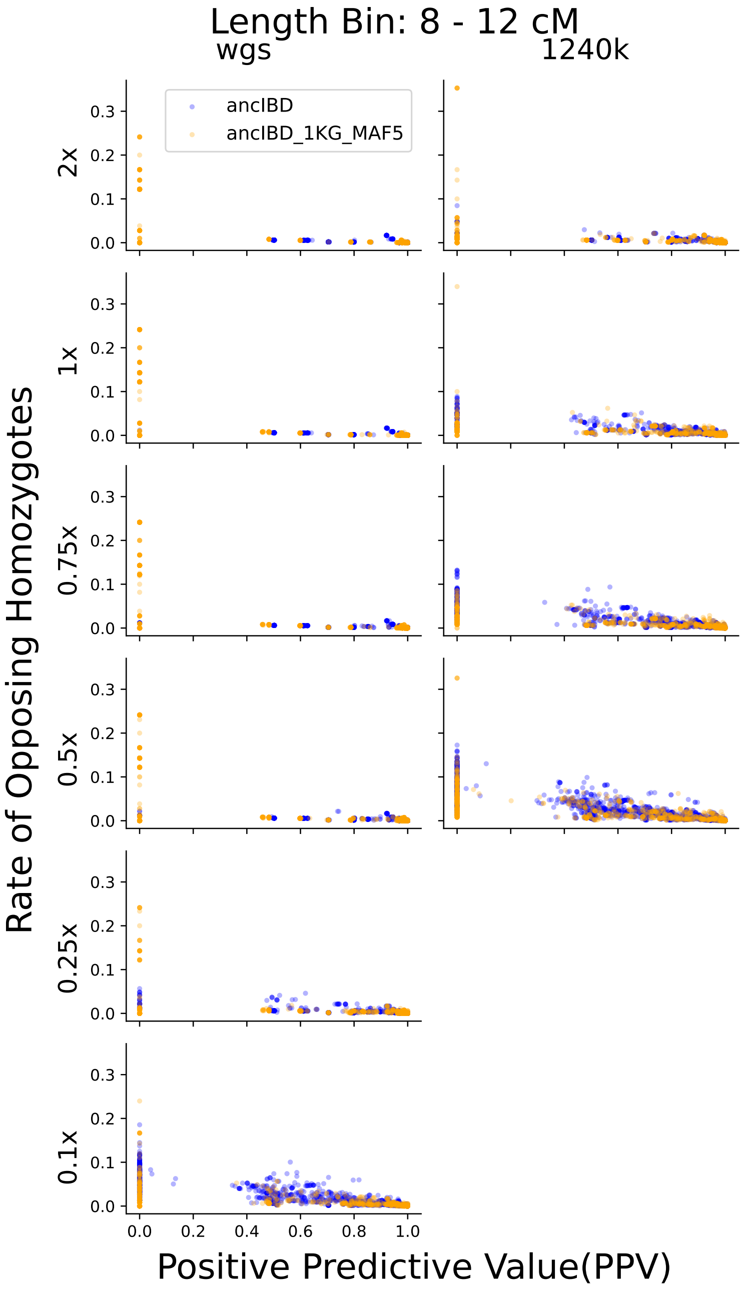

Supplementary Fig.13: Rate of Opposing Homozygotes for called segments in length bin 8-12cM.

of constant size (see e.g. [Ringbauer et al., 2021, Fernandes et al., 2021]).

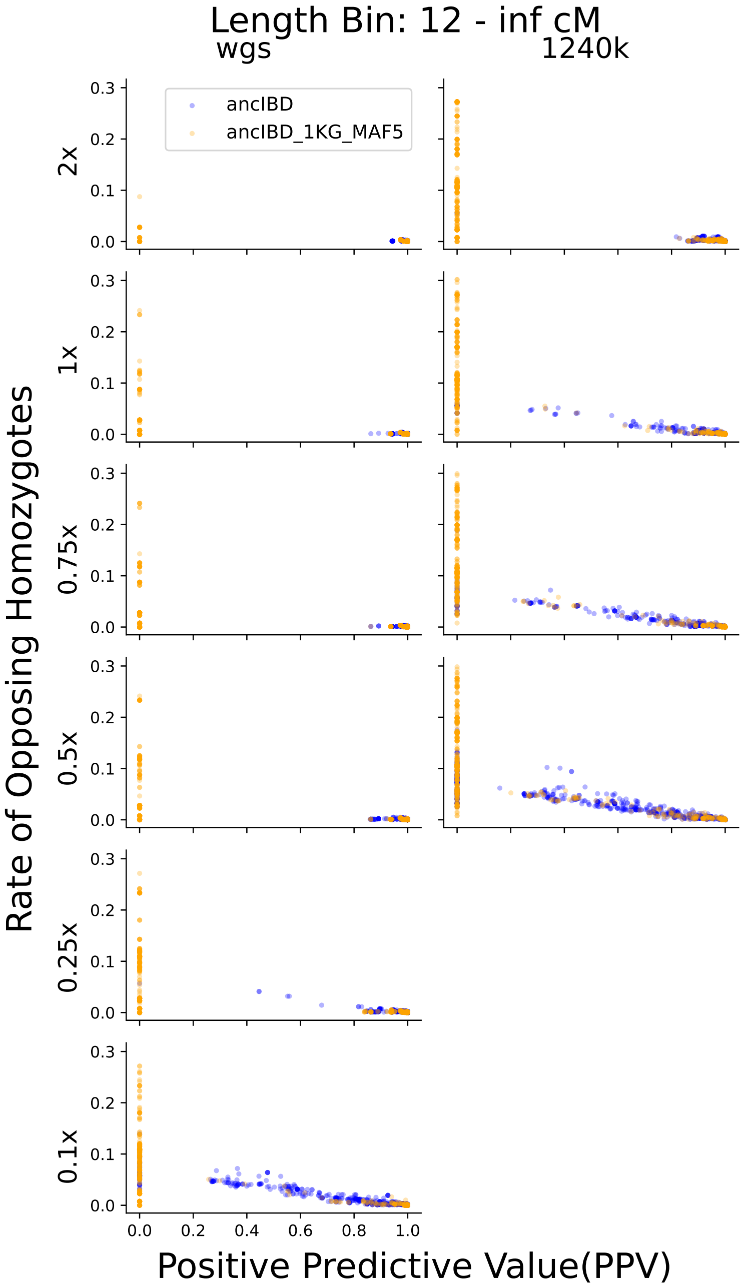

Supplementary Fig.14: Rate of Opposing Homozygotes for called segments in length bin ⩾12cM.

Supplementary Note 8 Simulating IBD-sharing of biological relatives using PED-SIM

To gain insight into the number and length distribution of IBD blocks given various degrees of close biological relatedness, one can calculate the expected numbers of blocks falling into certain length classes [Ringbauer et al., 2021, see e.g.]. While these calculations accurately predict the expected IBD sharing, they do not address the natural biological variance around the expectation. Moreover, they rely on the assumption that recombination can be modeled as a Poisson process (i.e. measuring genomic distances in Morgan) and do not incorporate the biological process of recombination interference (i.e. recombination events are less clustered than expected) as well as sex-specific recombination maps (in humans, the average cross-over rate in females is about 1.6 times higher than in males, with substantial fine-scale variation [Bhérer et al., 2017]). Previous experiments revealed that for relatives beyond the second degree, these model violations have only little impact; however, these processes can significantly influence patterns of IBD segment sharing for close relatives [Caballero et al., 2019].

For these reasons, we utilized the software ped-sim (v1.0.6) to simulate shared IBD segments between relatives [Caballero et al., 2019]. For each degree up to sixth-degree relatedness, we simulated 100 pairs of individuals each, using the sex-specific genomic map of [Bhérer et al., 2017], and simulating all autosomes with the recombination interference model [Campbell et al., 2015] incorporated into PED-SIM.

To compare with the IBD segment distribution in empirical ancient relatives, we applied the same IBD segment filter that we applied to post-process ancIBD output, i.e. we merged adjacent IBD segments, such as occurring when there is a switch between IBD1 and IBD2 states that are output as separate segments in ped-sim, into a single contiguous one and we removed IBD segments that have a density of 1240k SNP less than 220 per centimorgan. We note that the latter filter removes chromosomes 19 and 22 in case they are completely in IBD (e.g. in parent-offspring). One remaining caveat is that the sex-specific map of Bhérer et al. [2017]) used for ped-sim simulations sums up to 3346.3 cm (when sex-averaged), while the deCODE map [Kong et al., 2010] used for empirical IBD calls sums up to a total length of 3537.0 cm. However, we believe that the benefit of running simulations with a sex-specific map justifies this slight mismatch of total map lengths.

We visualize the simulated IBD sharing in Fig. 3b. We note that we simulated both ancestral relationships (e.g. parents and grandparents) and also relationships via full sibs (e.g. full sibs themselves or uncle/aunts). These two relationship types can have differ-

ent distributions of IBD lengths for the same degree of relatedness because the number of meiosis in relationships via full sibs is elevated by one while the number of shared haplotype ancestors is four instead of two.

Supplementary Note 9 Identifying relatives using pair-wise mismatch rates (PMR)

To compare IBD segment sharing in aDNA data to conventional methods to detect relatives, we calculated a relatedness estimate based on the pair-wise mismatch rate (PMR) that we describe below. This measure of a genome-wide average of allele-sharing is widely used in aDNA studies and underlies common methods to detect relatives in aDNA [Lipatov et al., 2015, Monroy Kuhn et al., 2018, e.g.].

We computed pairwise mismatch rates by randomly sampling one read for each individual at 1240k autosomal SNPs. For normalization, we estimated relatedness coefficients r as described in Kennett et al. [2017]:

$$r = 1 - \frac{x-b}{b}, \tag{1}$$

where x denotes the pairwise mismatch rate for that pair and b is the expected mismatch rate for two genetically identical individuals from the same population.

For each pair of individuals, we estimated b as the mean of the two intra-individual mismatch rates of each of the two individuals in the pair, which we computed by randomly sampling two reads at each position. To account for consanguinity (i.e. closely related parents), we corrected intra-individual mismatch rates by multiplying with a correction factor $1 - f_{\text{ROH}}$, where f_{ROH} denotes the fraction of an individual genome in ROH longer than 8 centimorgan. To estimate this fraction, we call ROH using the method hapROH using default parameters [Ringbauer et al., 2021].

Finally, we annotated up to third-degree relatives based on the inferred PMR-based relatedness estimates r (see Supplementary Fig.15). As cutoffs between the four states (unrelated, third, second, and first-degree relatives) we used the theoretical halfway boundaries of (0.09375, 0.18375, 0.375) between expected values of r (0, 0.125, 0.25, 0.5). Moreover, we filtered all pairs with relatedness estimates >0.75, which are indicating genetic duplicates.

We visualize the inferred PMR-based relatedness for all pairs of individuals with at least 500 cm of IBD segments longer than 12 cm in Supplementary Fig.15. We note that the few outliers are enriched for pairs of individuals with substantially different ances-

try, for which the approach of taking intra-individual diversity as a baseline for between individual diversity is evidently biased. This issue is a general problem of PMR-based methods, which can be overcome with IBD-segment-based analysis.

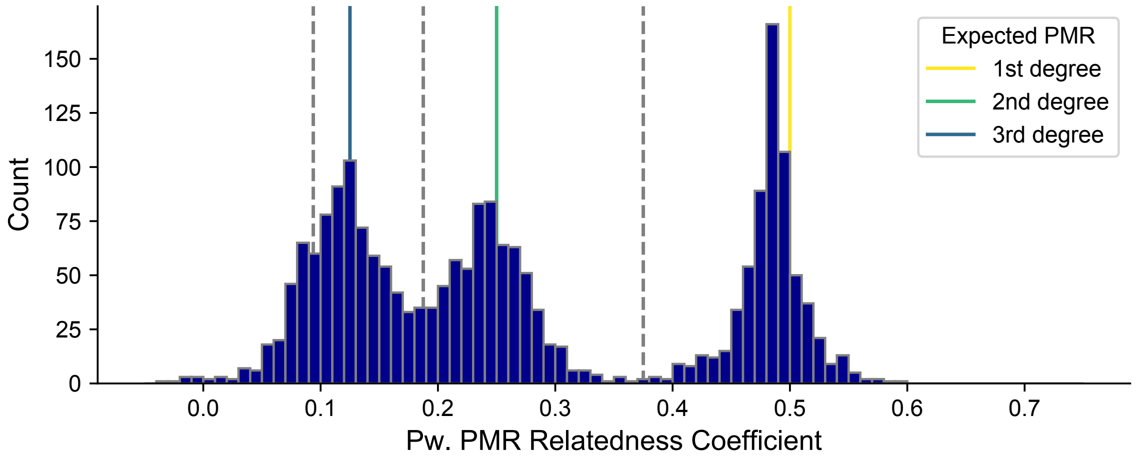

Supplementary Fig.15: Inferred PMR-based relatedness for empirical aDNA dataset. We calculated PMR-based relatedness based on Eq. 1 for all 2,089 pairs of ancient individuals where ancIBD identified at least 500 cm of IBD segments longer than 12 cm. We visualize the expected values for first, second, and third-degree relatives (0.5, 0.25, and 0.125, vertical lines), as well as the cutoffs halfway between expected values for various degrees of relatives (dotted vertical lines).

Supplementary Note 10 Comparison with other methods to detect IBD segments

In this section, we compare ancIBD's performance with other IBD callers designed for modern DNA data. To our knowledge, no dedicated IBD caller has been developed to be applied to human ancient DNA previously; however, the same fundamental principles of detecting IBD segments apply as for modern DNA data. Thus, methods designed for modern DNA data might extend to imputed low-coverage aDNA data.

IBDseq

IBDseq [Browning and Browning, 2013] is designed for whole genome sequencing data. It computes the likelihood ratio of IBD and non-IBD for each biallelic marker and then sums them to find long stretches of IBD regions. For applying IBDseq to imputed data, we filtered imputed variants to MAF >1% and imputation INFO score >0.8 (the same filtering as performed in Allentoft et al. [2022]). We merged the four ancient samples with 503 diploid samples from the 1000 Genome Project labeled as belonging to the super population EUR because IBDseq relies on population allele frequencies estimated from input samples. We found that for long segments (>12cM) and high coverage, both ancIBD and IBDseq perform equally well. In addition, compared with ancIBD, IBDseq has higher power in detecting intermediate segments (8-12cM) at higher coverage. However, IBDseq's precision quickly drops below an acceptable level for low coverages that are typical for most aDNA data (Supplementary Fig.16), especially for 1240k data. Additionally, we also tried to filter imputed variants further to only keep transversion sites to mitigate the effect of aDNA damage, however, we found that this filtering has only negligible effects on IBDseq's precision and recall (Supplementary Fig.17).

GERMLINE and GERMLINE 2

Both GERMLINE [Gusev et al., 2009] and GERMLINE 2 [Nait Saada et al., 2020] rely on accurate phasing as they take a seed-and-extend approach to search for identical haplotypes between two samples. For GERMLINE, we used the same SNP filtering as for IBDseq described above. We attempted to tune default parameters to accommodate the noisy nature of imputed aDNA data (e.g, turn on the '-g_extend' option recommended for noisy data, allowing up to 10 mismatch homozygous and heterozygous markers per slice); however, we could not identify any setting that enabled GERMLINE

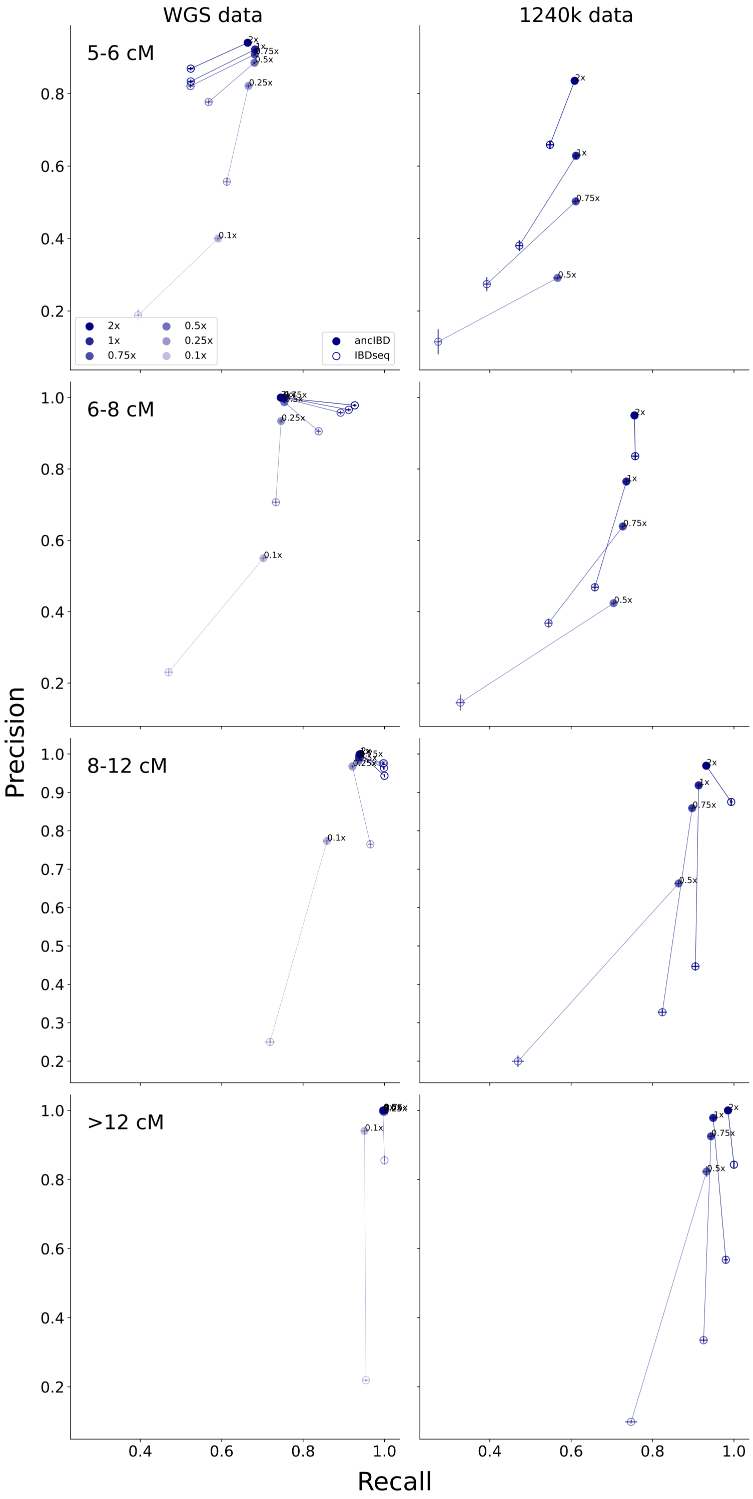

Supplementary Fig.16: Precision and recall of ancIBD and IBDseq at various length bins and coverages. We applied IBDseq as described above and compared its precision and recall with ancIBD at various coverages and IBD length bins. For each coverage, we report the average precision and recall of each length bin across 50 independent replicates. The error bar represents ±SE of the estimated precision and recall.

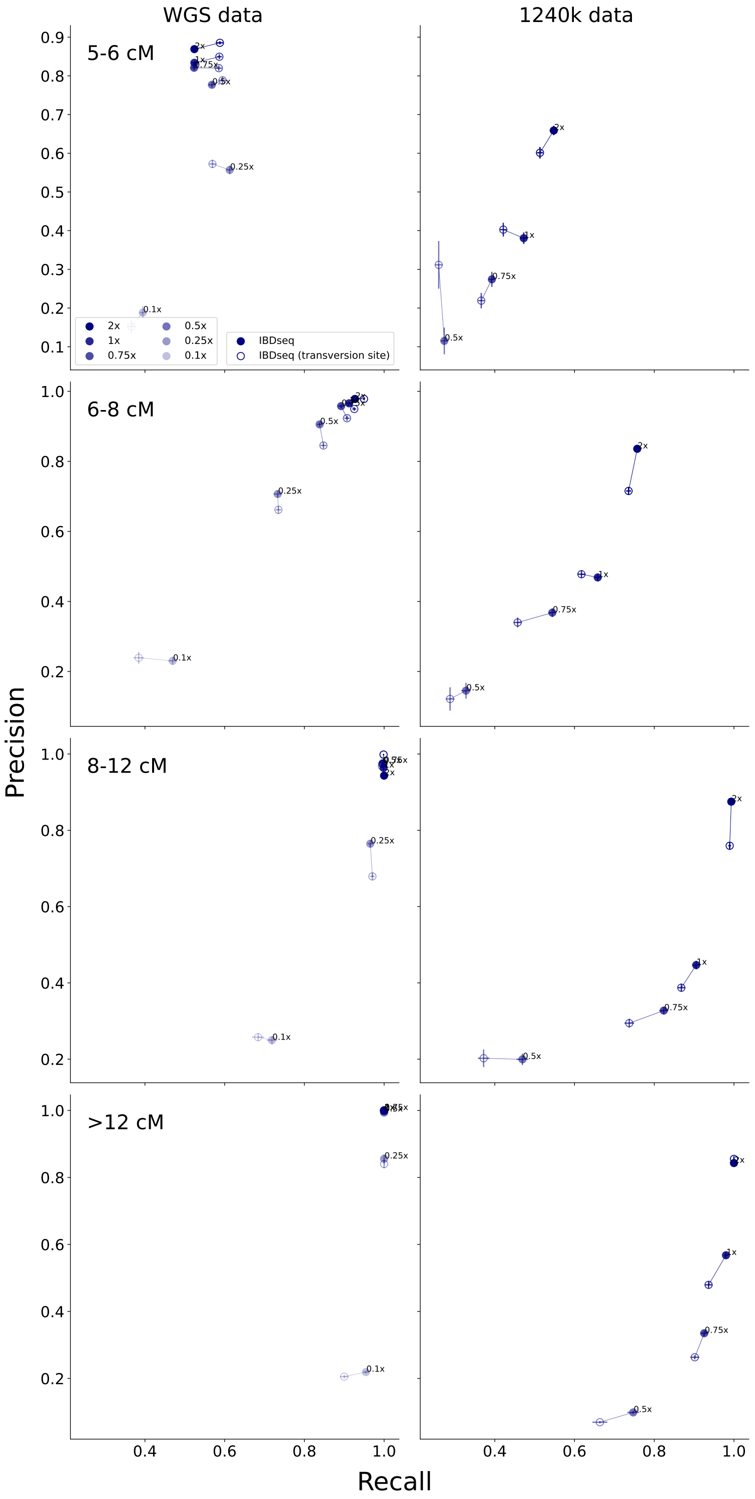

Supplementary Fig.17: Precision and recall of IBDseq with and without filtering transition SNPs. For each coverage, we report the average precision and recall of each length bin across 50 independent replicates. The error bar represents ±SE of the estimated precision and recall.

or GERMLINE 2 to detect any IBD segments among the test samples. Having effectively zero power is most likely due to the relatively high switch error rates in aDNA data imputed with modern reference panels (Tab. S2), which is an order of magnitude higher than what is attained for modern DNA phased with biobank scale reference panel [Sousa da Mota et al., 2023, Rubinacci et al., 2021].

hapIBD

Similar to GERMLINE, hapIBD [Zhou et al., 2020] requires phased genotypes. We used the same SNP filtering as for IBDseq and adjusted hapIBD's default parameters to allow more mismatches (min-seed=0.1, min-extend=0.05, max-gap=500000, where the default values for the three parameters are 2.0, 1.0, 1000, respectively). Despite those attempts, hapIBD's power remains very low and the detected segments tend to be highly fragmented (Supplementary Fig.18), making it generally not applicable for imputed aDNA data.

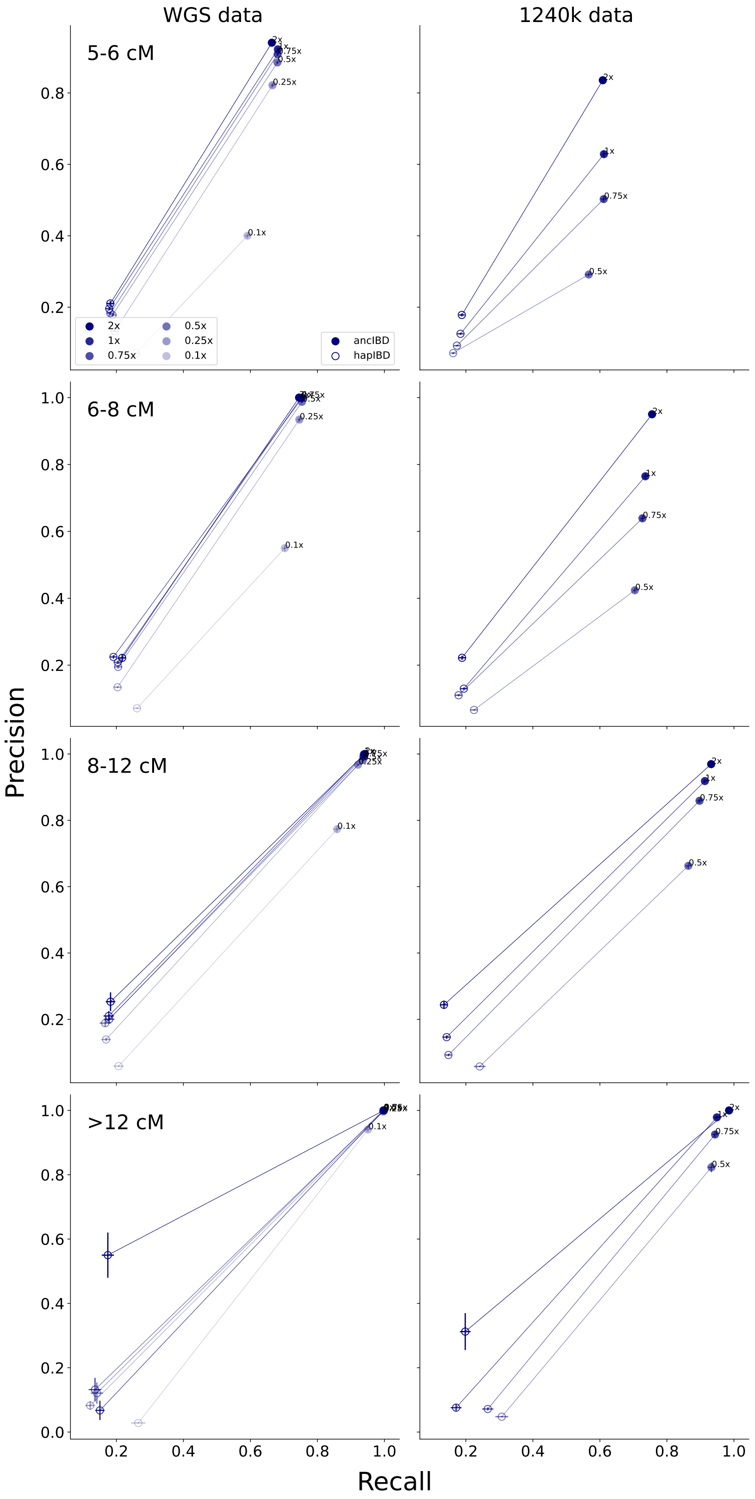

Supplementary Fig.18: Precision and recall of hapIBD at various length bins and coverages. We applied hapIBD as described above at various coverages. For each coverage, we report the average precision and recall of each length bin across 50 independent replicates. The error bar represents ±SE of the estimated precision and recall.

Supplementary Note 11 Other Supplementary Figures

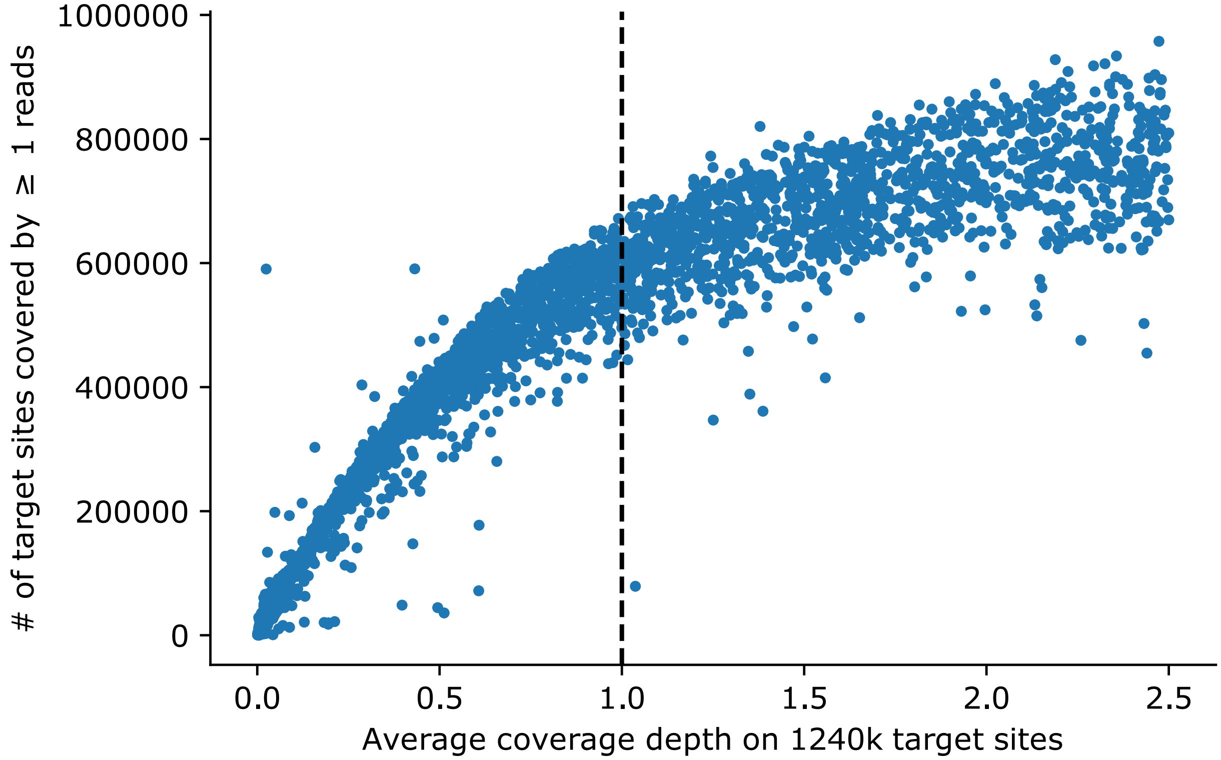

Supplementary Fig.19: Relationship between number of sites covered by ⩾ 1 reads and average coverage depth on 1240k SNP sites. The plot shows the average coverage depth and the number of sites covered for 1240k samples from AADR (release v54.1). The recommended coverage cutoff (1x) is indicated by a black vertical dashed line. Only samples with less than 2.5x coverage are depicted.

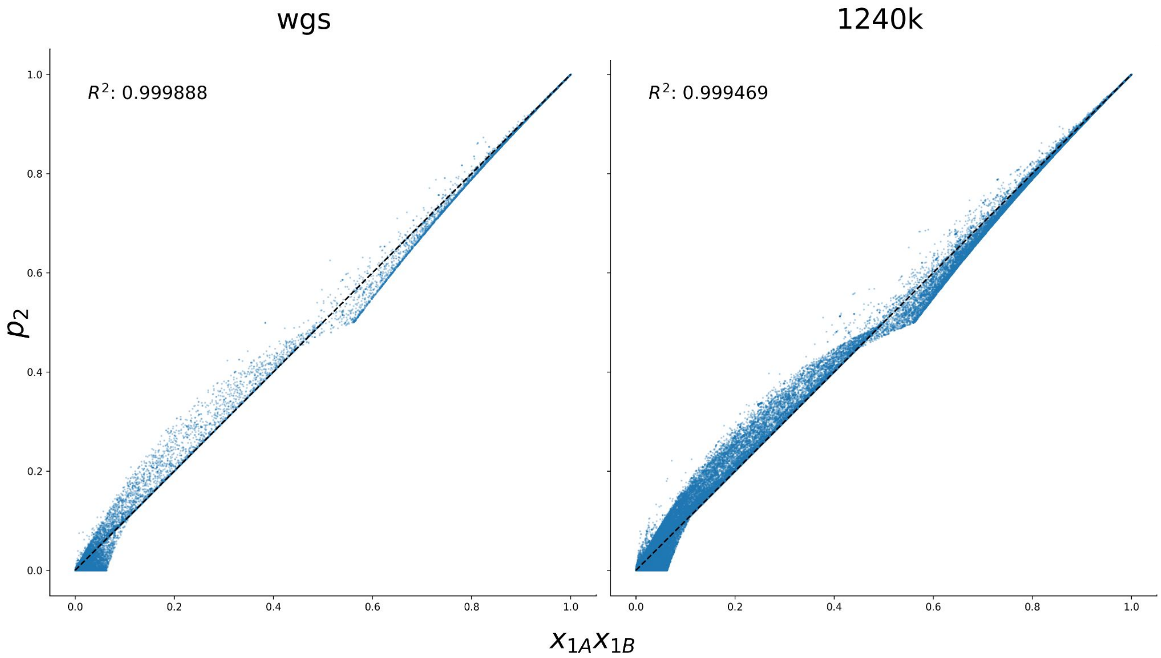

Supplementary Fig.20: Validity of approximating diploid genotype probabilities as the product of haplotype probabilities. As described in the main text, we approximate $P(\mathbf{g}|D)$ as the product of the four probabilities of each of the haplotypes (1A, 1B) and (2A, 2B) being reference or alternative. Here we check the validity of this approximation by plotting p_2 against $x_{1A}x_{1B}$, where p_2 is the GLIMPSE-estimated genotype probability of being homozygous alternative alleles. The data points come from all the variants on chr1 in the 1000 Genome reference panel. The figure shows the result of I2105 downsampled to 1x. The coefficient of determination (calculated from sklearn.metrics.r2_score) is indicated in the upper left corner.

References

Morten E Allentoft, Martin Sikora, Alba Refoyo-Martínez, Evan K Irving-Pease, Anders Fischer, William Barrie, Andrés Ingason, Jesper Stenderup, Karl-Göran Sjögren, Alice Pearson, et al. Population genomics of stone age eurasia. *bioRxiv*, 2022.

Claude Bhérer, Christopher L Campbell, and Adam Auton. Refined genetic maps reveal sexual dimorphism in human meiotic recombination at multiple scales. *Nature communications*, 8(1):14994, 2017.

Arjun Biddanda, Daniel P Rice, and John Novembre. A variant-centric perspective on geographic patterns of human allele frequency variation. *Elife*, 9:e60107, 2020.

Brian L Browning and Sharon R Browning. A fast, powerful method for detecting identity by descent. *The American Journal of Human Genetics*, 88(2):173–182, 2011.

Brian L Browning and Sharon R Browning. Detecting identity by descent and estimating genotype error rates in sequence data. *The American Journal of Human Genetics*, 93(5): 840–851, 2013.

Madison Caballero, Daniel N Seidman, Ying Qiao, Jens Sannerud, Thomas D Dyer, Donna M Lehman, Joanne E Curran, Ravindranath Duggirala, John Blangero, Shai Carmi, et al. Crossover interference and sex-specific genetic maps shape identical by descent sharing in close relatives. *PLoS Genetics*, 15(12):e1007979, 2019.

Christopher L Campbell, Nicholas A Furlotte, Nick Eriksson, David Hinds, and Adam Auton. Escape from crossover interference increases with maternal age. *Nature communications*, 6(1):6260, 2015.

1000 Genomes Project Consortium et al. A global reference for human genetic variation. *Nature*, 526(7571):68–74, 2015.

Petr Danecek, Adam Auton, Goncalo Abecasis, Cornelis A Albers, Eric Banks, Mark A DePristo, Robert E Handsaker, Gerton Lunter, Gabor T Marth, Stephen T Sherry, et al. The variant call format and vcftools. *Bioinformatics*, 27(15):2156–2158, 2011.

Daniel M Fernandes, Kendra A Sirak, Harald Ringbauer, Jakob Sedig, Nadin Rohland, Olivia Cheronet, Matthew Mah, Swapan Mallick, Iñigo Olalde, Brendan J Culleton, et al. A genetic history of the pre-contact caribbean. *Nature*, 590(7844):103–110, 2021.

Alexander Gusev, Jennifer K Lowe, Markus Stoffel, Mark J Daly, David Altshuler, Jan L Breslow, Jeffrey M Friedman, and Itsik Pe'er. Whole population, genome-wide mapping of hidden relatedness. *Genome research*, 19(2):318–326, 2009.

Ruoyun Hui, Eugenia D'Atanasio, Lara M Cassidy, Christiana L Scheib, and Toomas Kivisild. Evaluating genotype imputation pipeline for ultra-low coverage ancient genomes. *Scientific Reports*, 10(1):1–8, 2020.

Douglas J Kennett, Stephen Plog, Richard J George, Brendan J Culleton, Adam S Watson, Pontus Skoglund, Nadin Rohland, Swapan Mallick, Kristin Stewardson, Logan Kistler, et al. Archaeogenomic evidence reveals prehistoric matrilineal dynasty. *Nature communications*, 8(1):14115, 2017.

Augustine Kong, Gudmar Thorleifsson, Daniel F Gudbjartsson, Gisli Masson, Asgeir Sigurdsson, Aslaug Jonasdottir, G Bragi Walters, Adalbjorg Jonasdottir, Arnaldur Gylfason, Kari Th Kristinsson, et al. Fine-scale recombination rate differences between sexes, populations and individuals. *Nature*, 467(7319):1099–1103, 2010.

Heng Li. A statistical framework for snp calling, mutation discovery, association mapping and population genetical parameter estimation from sequencing data. *Bioinformatics*, 27 (21):2987–2993, 2011.

Mikhail Lipatov, Komal Sanjeev, Rob Patro, and Krishna R Veeramah. Maximum likelihood estimation of biological relatedness from low coverage sequencing data. *BioRxiv*, page 023374, 2015.

Swapan Mallick, Adam Micco, Matthew Mah, Harald Ringbauer, Iosif Lazaridis, Iñigo Olalde, Nick J Patterson, and David E Reich. The allen ancient dna resource (aadr): A curated compendium of ancient human genomes. *bioRxiv*, pages 2023–04, 2023.

Iain Mathieson, Songül Alpaslan-Roodenberg, Cosimo Posth, Anna Szécsényi-Nagy, Nadin Rohland, Swapan Mallick, Iñigo Olalde, Nasreen Broomandkhoshbacht, Francesca Candilio, Olivia Cheronet, et al. The genomic history of southeastern europe. *Nature*, 555(7695):197–203, 2018.

José Manuel Monroy Kuhn, Mattias Jakobsson, and Torsten Günther. Estimating genetic kin relationships in prehistoric populations. *PloS one*, 13(4):e0195491, 2018.

Juba Nait Saada, Georgios Kalantzis, Derek Shyr, Fergus Cooper, Martin Robinson, Alexander Gusev, and Pier Francesco Palamara. Identity-by-descent detection across

487,409 british samples reveals fine scale population structure and ultra-rare variant associations. *Nature communications*, 11(1):1–15, 2020.

Vagheesh M Narasimhan, Nick Patterson, Priya Moorjani, Nadin Rohland, Rebecca Bernardos, Swapan Mallick, Iosif Lazaridis, Nathan Nakatsuka, Iñigo Olalde, Mark Lipson, et al. The formation of human populations in south and central asia. *Science*, 365(6457):eaat7487, 2019.

Peter Ralph and Graham Coop. The geography of recent genetic ancestry across europe. *PLoS biology*, 11(5):e1001555, 2013.

Harald Ringbauer, John Novembre, and Matthias Steinrücken. Parental relatedness through time revealed by runs of homozygosity in ancient dna. *Nature communications*, 12(1):5425, 2021.

Simone Rubinacci, Diogo M Ribeiro, Robin J Hofmeister, and Olivier Delaneau. Efficient phasing and imputation of low-coverage sequencing data using large reference panels. *Nature Genetics*, 53(1):120–126, 2021.

Daniel N Seidman, Sushila A Shenoy, Minsoo Kim, Ramya Babu, Ian G Woods, Thomas D Dyer, Donna M Lehman, Joanne E Curran, Ravindranath Duggirala, John Blangero, et al. Rapid, phase-free detection of long identity-by-descent segments enables effective relationship classification. *The American Journal of Human Genetics*, 106(4):453–466, 2020.

Bárbara Sousa da Mota, Simone Rubinacci, Diana Ivette Cruz Dávalos, Carlos Eduardo G. Amorim, Martin Sikora, Niels N Johannsen, Marzena H Szmyt, Piotr Włodarczak, Anita Szczepanek, Marcin M Przybyła, et al. Imputation of ancient human genomes. *Nature Communications*, 14(1):3660, 2023.

Shamam Waldman, Daniel Backenroth, Éadaoin Harney, Stefan Flohr, Nadia C Neff, Gina M Buckley, Hila Fridman, Ali Akbari, Nadin Rohland, Swapan Mallick, et al. Genome-wide data from medieval german jews show that the ashkenazi founder event pre-dated the 14th century. *Cell*, 185(25):4703–4716, 2022.

Anthony Wilder Wohns, Yan Wong, Ben Jeffery, Ali Akbari, Swapan Mallick, Ron Pinhasi, Nick Patterson, David Reich, Jerome Kelleher, and Gil McVean. A unified genealogy of modern and ancient genomes. *Science*, 375(6583):eabi8264, 2022.

James A Fellows Yates, Thiseas C Lamnidis, Maxime Borry, Aida Andrades Valtueña, Zandra Fagernäs, Stephen Clayton, Maxime U Garcia, Judith Neukamm, and Alexan-

der Peltzer. Reproducible, portable, and efficient ancient genome reconstruction with nf-core/eager. *PeerJ*, 9:e10947, 2021.

Ying Zhou, Sharon R Browning, and Brian L Browning. A fast and simple method for detecting identity-by-descent segments in large-scale data. *The American Journal of Human Genetics*, 106(4):426–437, 2020.
